# 6D Building information modelling (BIM) for demolition waste management of semi-submersible wind turbines

**DOI:** 10.1038/s41598-026-52117-2

**Published:** 2026-06-02

**Authors:** Sakdirat Kaewunruen, Konstantinos Frantzezos, Yi-Hsuan Lin, Charalampos Baniotopoulos, Jian Zhang

**Affiliations:** 1 Department of Civil Engineering, University of Birmingham, Birmingham, B15 2TT UK; 2 Department of Civil Engineering, Leibniz University Hannover, Welfengarten 1, 30167 Hannover, Germany; 3 Faculty of Mechanical Engineering, Jiangsu University of Science and Technology, Dantu District, Zhenjiang City, 212100 Jiangsu Province P.R. China

**Keywords:** Building information modelling (BIM), Floating wind turbines (FWTs), Offshore wind decommissioning, Circular economy (CE), Sustainability, Energy science and technology, Engineering, Environmental sciences

## Abstract

The rapid global expansion of offshore wind is matched by an emerging wave of decommissioning. By the 2030s, over 30 GW of Europe’s capacity will reach end-of-life (EoL), rising to 40 million tonnes of material waste globally by 2050, with blades alone contributing approximately 325,000 tonnes of waste per year in Europe if not effectively recovered. This shift presents a risk of waste accumulation and an opportunity to embed circular economy (CE) practices into infrastructure renewal. To address this challenge, this study develops a BIM-based framework for dismantling a semi-submersible floating wind turbine (FWT), combining 3D modelling, 4D time sequencing and component-level inventories. The analysis adopts three condition-based scenarios — intact, minor and major damage — reflecting the real uncertainty at EoL, where exposure to marine environments can produce highly variable outcomes. This structure allows engineers to assess recovery routes in a realistic manner, from high-value reuse to advanced recycling. Our new findings demonstrate that Scenario 1 enables reuse and delivers the lowest cost, time and CO₂e impacts, while enabling the highest-value CE applications. Scenario 2 introduces moderate repair burdens due to processing requirements and Scenario 3 is dominated by energy-intensive processes. By linking those results to cumulative inventory, the study provides a replicable digital catalog that captures component fate across scenarios. The creation of material passports / inventories provides traceability of resources, highlights landfill avoidance and supports market preparation for secondary materials. For engineers, the framework offers a reproducible, inspection-driven decision-support tool that links component conditions to dismantling schedules and CE pathways, informing planning and procurement in upcoming decommissioning projects of the offshore wind industry.

## Introduction

The offshore wind industry is entering a decisive end-of-life (EoL) phase that will reshape how renewable energy infrastructure is managed. By the 2030s, more than 30 GW of installed offshore wind capacity in Europe alone will require decommissioning^1^ and by 2050, an estimate around 40 million tonnes of turbine materials worldwide will reach EoL^2^. Steel, which constitutes 70–80% of turbine mass, is readily recyclable, yet composite blades pose a severe burden with projections suggesting 325,000 tonnes of composite blade waste to be generated annually in Europe by 2050^3–5^. Conventional practices remain costly and materially wasteful, especially for composite-rich components and submerged infrastructure, risking unnecessary carbon and value loss^6,7^. Without structured strategies, decommissioning risks reproducing linear component disposal models that undermine circular economy (CE) ambitions.

Digitalisation provides new opportunities to address these issues. While Building Information Modelling (BIM) is established across design and construction, its application to EoL planning for offshore wind is limited and links to CE decision-making are rarely operationalised^8,9^. Through its ability to visualise workflows and embed material data, BIM can generate component-specific material passports, ensuring that resources are tracked, quantified and directed to CE-aligned pathways.

**Fig. 1:**
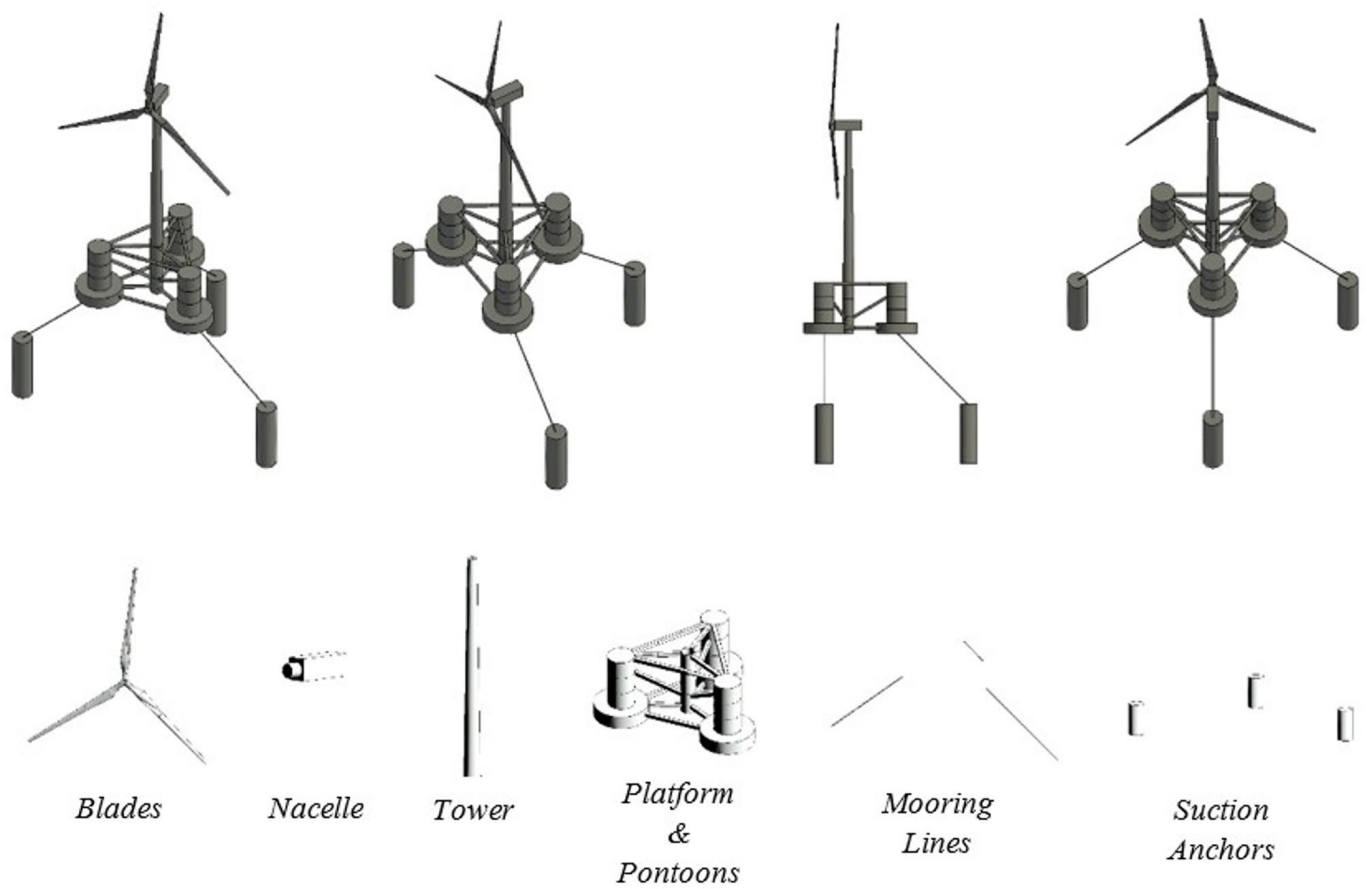
Semi-submersible wind turbine and components in Autodesk Revit.

This study responds to the gaps by developing a BIM-centred decommissioning approach for a semi-submersible floating wind turbine (FWT) modelled in Autodesk Revit (Fig. 1), coupling component-level digital modelling with scenario-based calculations of cost, time and CO₂e to inform high-value recovery pathways. The framework presented here derives masses from BIM geometry, routes components through three EoL condition scenarios, links the results with 4D sequencing in Navisworks and introduces time-cost-carbon outputs and post-decommissioning inventories.

The BIM framework for the FWT was developed to capture key system-specific parameters for the principal structural and functional components. These include the blades, nacelle, tower, floating platform, pontoons, mooring lines, and suction anchors (Fig. 1). The modelling approach emphasises component-level granularity, enabling the systematic representation of material composition, geometric characteristics, and assembly relationships. The treatment, assumptions, and characterisation of each component are described in detail within the methodology section to ensure transparency and reproducibility. To maintain analytical clarity and focus, the BIM model was intentionally simplified by excluding internal mechanical and electrical subsystems. These elements were considered beyond the scope of the present study, as they do not materially influence the dominant material flows or waste generation associated with decommissioning. Instead, the analysis prioritises the primary structural components, which account for the majority of mass and, consequently, the most significant opportunities for CE strategies, including reuse, recycling, and material recovery.

By doing so, this research considers whether BIM can act as a decision-support tool for decommissioning planning, what component-specific strategies best align with CE outcomes and how quantitative assessment of cost, time and carbon emissions can inform more sustainable offshore wind policy and practice.

The aim of this study is to establish a digital framework that demonstrates how BIM can be applied to FWT decommissioning, enabling data-driven planning and quantifying time, cost and carbon impacts while advancing CE practices. In this paper, we establish 6D BIM model of a semi-submersible floating wind turbine using Autodesk Revit and then derive component-level material quantities as a digital product/material passport. Based on industry consultation, three EoL condition scenarios are considered: (i) perfect, (ii) minor and (iii) major damage across key components in order to assess variability in decommissioning outcomes. The BIM is capable of quantifying cost, time and CO₂e impacts of decommissioning stages using secondary data sources linked to the BIM-derived material passport. The BIM integrates 4D time sequencing in Navisworks for visual simulation of phased deconstruction. This study showcases a new development of component-level inventories, identifying EoL applications that embed CE principles applicable and scalable to offshore wind farms.

## Literature review

The European Commission^10^ and HM Government^11^ have both established long-term decarbonisation strategies to achieve net-zero greenhouse gas emissions by 2050. These policy frameworks aim to mitigate climate change while promoting long-term societal sustainability across industrial sectors. In response, an increasing body of research has investigated approaches to reduce greenhouse gas emissions and improve resource efficiency within the wind energy sector, with particular emphasis on end-of-life (EoL) management and circular economy (CE) strategies.

Existing studies have primarily focused on developing and evaluating EoL pathways for onshore wind turbines. For instance, Kramer et al.^12^ quantified potential EoL scenarios through expert-based assessments combined with material flow modelling, providing insights into the potential for material recovery. Similarly, Gast et al.^13^ applied circularity indices to assess material flows and energy consumption across the lifecycle of wind turbine components, highlighting opportunities for improved resource efficiency. In addition, He et al.^14^ investigated recycling technologies for wind turbine blades, particularly targeting the recovery of high-value materials to enhance circular economy performance and reduce environmental impacts. Collectively, these studies demonstrate the growing interest in circularity and resource recovery within the wind energy sector; however, they largely concentrate on onshore wind turbine systems and component-level solutions.

In contrast, offshore wind turbines present significantly greater technical, logistical, and economic complexity compared with onshore systems. Offshore wind infrastructure involves additional components, including floating platforms, mooring systems, and subsea anchoring structures, which introduce greater material diversity and decommissioning challenges. Furthermore, material compositions differ substantially; for example, onshore wind turbine foundations are typically constructed from reinforced concrete^13^, whereas offshore structures are predominantly fabricated from structural steel to withstand harsh marine environments. From an economic perspective, offshore wind turbines also require higher costs and longer timeframes for installation and decommissioning due to offshore logistics, specialised vessels, and operational constraints.

Despite these differences, limited research has addressed integrated EoL strategies specifically for offshore wind infrastructure. Therefore, there remains a clear research gap in developing comprehensive and system-level EoL management approaches tailored to offshore wind turbines, which this study aims to address. A summary of the comparison between onshore and offshore wind turbines described above is presented in Table 1.

**Table 1:** Comparison between onshore and offshore wind turbines^10–16^.

Aspect	Onshore wind turbines	Offshore wind turbines
Installation location	Land-based environments	Marine environments (fixed-bottom or floating)
Structural components	Tower, nacelle, blades, and concrete foundation	Tower, nacelle, blades, steel foundation, floating platforms, mooring systems, and subsea cables
Foundation type	Reinforced concrete foundations	Monopile, jacket, gravity-based, or floating foundations (primarily steel)
Material composition	Higher proportion of concrete and composite materials	Higher proportion of structural steel and corrosion-resistant materials
Installation complexity	Relatively simple logistics and transportation	Requires specialised vessels, offshore cranes, and marine operations
Decommissioning complexity	Easier dismantling and site restoration	Complex decommissioning involving underwater structures and offshore logistics
Cost	Lower installation and decommissioning costs	Higher installation and decommissioning costs
Maintenance access	Easy access via road transport	Limited access and weather-dependent maintenance operations
Lifetime	Typically 20–25 years	Typically 20–40 years
Recycling infrastructure	More established recycling pathways	Limited recycling infrastructure and experience
Environmental challenges	Land use, noise, and visual impact	Marine ecosystem impacts, corrosion, and harsh environmental conditions
Technical complexity	Lower technical complexity	Higher technical and operational complexity

### Offshore wind infrastructure at EoL

Offshore wind power is a cornerstone of global decarbonisation strategies, with deployment expanding into deeper waters via floating platforms. Semi-submersible foundations have emerged as one of the most commercially viable solutions, offering stability, modularity and tow-to-port maintenance capability^17,18^. Despite significant growth in deployment, EoL strategies for such infrastructure remain underdeveloped and studies estimate that approximately 40 million tonnes of wind turbine materials will reach EoL by 2050^2^ with regional contributions estimated at 40% from China, 25% from Europe, 16% from the United States, and 19% from the rest of the world. Many early offshore projects are commissioned without robust decommissioning frameworks while regulatory guidance often remains generic^15^.

Floating systems add complexity to EoL operations through submerged components while remaining cost effective^19^. Topham et al.^20^ address the little scholarly and industry attention that decommissioning floating structures receive. While steel, which constitutes 70–80% of turbine mass^6^, is readily recyclable, composite blades present technical and economic barriers estimated to generate 325,000 tonnes of waste annually in Europe by 2050^4,5^. Ineffective EoL strategies — such as landfilling blades or scrapping structures without material recovery — significantly increase lifecycle carbon footprints^1,21^. There is currently no standardised approach to simulating or optimising the deconstruction of FWT, highlighting a critical research gap.

### Semi-submersible floating wind turbine

Semi-submersible platforms typically include a central column supporting the turbine tower, three or more buoyant columns linked by pontoons and mooring lines with suction anchors^22^. Their modular construction enables full retrieval and component separation, aligning well with digital modelling approaches such as BIM for material tracking and EoL planning^8,23^. Extensive use of steel supports high-value recycling routes^20^, while detachable modules enable repurposing^24^.

However, their scale, irregular geometries and submerged infrastructure require advanced planning to dismantle efficiently while maximising recovery. Ciuriuc et al.^18^ note that the structural separability of semi-submersibles lends itself to BIM-enabled deconstruction modelling, yet practical case studies are almost non-existent. This disconnection between technical feasibility and applied practice highlights the need to situate semi-submersible decommissioning within broader waste management and CE frameworks, where digital tools like BIM can bridge the gap between design-stage potential and EoL implementation.

### Demolition waste management (DWM) and CE in offshore wind

Framing semi-submersible turbines within this wider context reveals the scale of the offshore decommissioning challenge. By 2050, an estimated 40 million tonnes of wind turbine components will reach EoL, much from offshore sources^7^. Yet waste management practices remain linear prioritising disposal over recovery and lack integration with CE models^21^. Additionally, research to date has primarily focused on onshore turbines, leaving floating systems comparatively underexplored^15^.

A recurring limitation in past studies is the fragmented treatment of offshore structures as isolated waste streams rather than interconnected systems with little emphasis on their decommissioning pathways. Moreover, while some research explores recycling technologies, less attention has been given to digital tools that could model these complex interdependencies in advance^18^. Addressing this gap is critical, as society increasingly demands decommissioning strategies that balance environmental protection, cost efficiency and material recovery.

For semi-submersibles, challenges extend to recovering and reusing mooring systems and submerged structures. Successful recovery depends on early integration of spatial disassembly modelling and identification of viable secondary applications. Yet the literature reveals that such integration — particularly through digital tools like BIM — is rarely implemented in offshore demolition^9^. This gap highlights the urgent need for research that unites CE principles with digital modelling.

### BIM for EoL planning and circular design

BIM has become a basis of architecture, engineering and construction (AEC) supporting design coordination, asset management and lifecycle costing. However, its use in offshore wind decommissioning is rare despite clear potential^8^. BIM offers granular control over demolition workflows by allowing models to be segmented into components, annotated with material and condition data and linked to cost, schedule and carbon metrics^9^.

In Revit-based workflows, a semi-submersible can be modelled as discrete families each with assigned material properties. Such models can then be phased to simulate disassembly sequences, with datasets exported to platforms like Navisworks for 4D scheduling and cost/carbon analysis^25^. This approach supports CE principles by enabling planners to evaluate reuse, repair, or recycling pathway in advance, aligning decommissioning with CE before physical dismantling.

Despite these capabilities, Velenturf^1^ and Woo and Whale^7^ argue that most current decommissioning practices rely on fragmented data leading to missed recovery opportunities. Even when BIM is used, sustainability metrics and CE frameworks are often absent^26^. While Digital Twins promise advanced lifecycle monitoring, their application to EoL stages in offshore wind remains largely theoretical and extends beyond the scope of this study.

### Knowledge gaps

While digital deconstruction is emerging in the built environment, it has yet to be applied in a structured way to floating offshore systems^8^. Research that does exist often treats waste management, structural modelling and CE as discrete domains, creating disciplinary silos that prevent a holistic approach. No existing studies, standards and practices provide a comprehensive, replicable framework linking BIM-based modelling, component-specific EoL strategies and CE principles for a semi-submersible FWT.

The literature highlights several gaps that this study aims to address:

1. Lack of integrated, BIM-enabled workflows for semi-submersible FWT decommissioning.
2. Minimal linkage between BIM-based disassembly planning and CE strategies in offshore contexts.
3. Limited practical modelling of component-level material flows, time, costs and carbon impacts to inform EoL decision-making.

This study responds by developing a BIM-centred DWM framework for a semi-submersible FWT. It models each component for condition-based EoL routing, integrates 4D scheduling and cost/carbon estimation and produces scenario-specific inventories to link recovered assets with viable CE applications. By doing so, it bridges methodological gaps in the literature and provides practical tools for industry as the first wave of floating wind decommissioning approaches. Table 2 summarises key studies on wind turbine end-of-life.

**Table 2:** Summary of key studies on offshore wind turbine EoL.

References	Focus area	Methodology	Key findings	Research gap
European Commission^10^; HM Government^11^	Decarbonisation policy	Policy frameworks	Net-zero targets by 2050 driving offshore wind turbines expansion	Limited guidance on EoL management
He et al.^14^	Blade recycling	Recycling technology assessment	Recovery of high-value composite materials	Focused on component-level solutions
Topham et al.^20^	Offshore decommissioning	Industry and academic review	Floating wind turbines decommissioning poorly addressed	Lack of practical case studies
Ciuriuc et al.^18^	Semi-submersible structures	BIM-enabled deconstruction concept	Semi-submersibles suitable for modular dismantling	Limited real-world implementation
Velenturf^1^	Offshore wind EoL sustainability	Lifecycle sustainability analysis	Poor EoL strategies increase carbon footprint	Fragmented decommissioning approaches
Woo and Whale^7^	Offshore wind waste	Waste management analysis	40 million tonnes reaching EoL by 2050	Lack of CE integration
Jia et al.^8^; Akbarieh et al.^9^	BIM for deconstruction	Digital modelling and 4D simulation	BIM enables material tracking and planning	Limited offshore wind application
Won and Cheng^26^	Lifecycle monitoring	Digital twin concepts	Potential for lifecycle optimisation	EoL application still theoretical

## Envisaged research methodologies/data sources

**Fig. 2:**
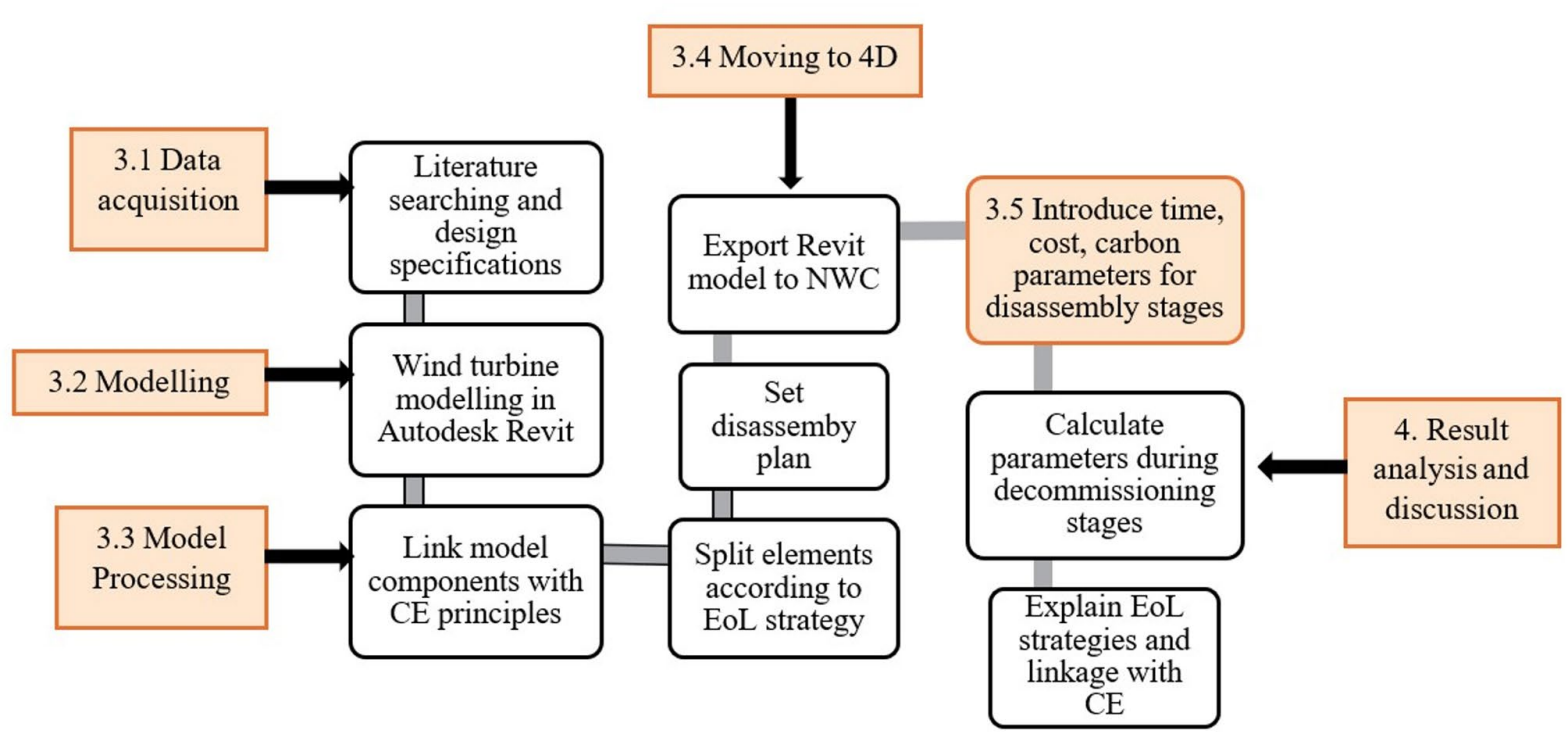
Methodology of our study.

This section covers the methodologies followed for the data collection, analysis and presentation for this study as shown in Fig. 2, centred on BIM to simulate and evaluate EoL strategies for a semi-submersible FWT.

The study will focus primarily on simulation and digital modeling, ensuring full compliance with the University of Birmingham’s ethical guidelines^27^. No primary human data will be collected.

### Research design and data sources

A qualitative, simulation-led research design has been adopted. The literature review is compiled from peer-reviewed journal articles, industry reports and technical guidelines sourced from databases such as ScienceDirect, SpringerLink and the UoB Library. Selection criteria prioritises relevance, credibility and novelty with sources addressing semi-submersible turbine design, BIM-based demolition planning, offshore waste management and CE strategies^18,22,28,29^. These inform both the technical modelling parameters and the sustainability framework underpinning the study.

Design specifications for the turbine model are derived from established floating wind references and documented in Appendix A^28–30^. The model is intentionally simplified by excluding internal mechanical and electrical systems, considering them beyond scope as they do not materially affect major waste flows. Their exclusion does not diminish the value of the findings but rather ensures that results are robust, reproducible and directly relevant to the most material aspects of decommissioning and CE outcomes.

### BIM-based modelling and component specification

**Fig. 3:**
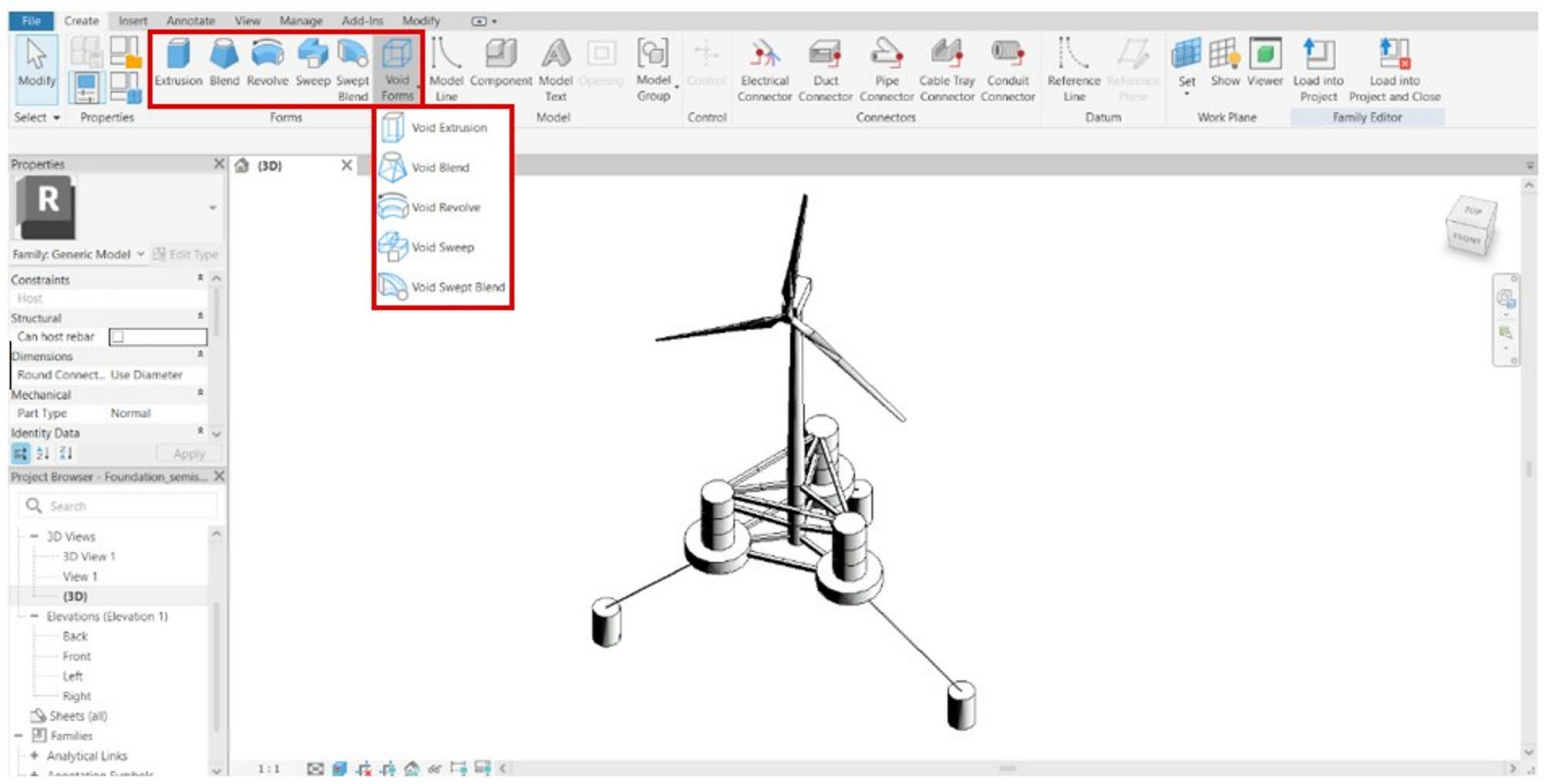
Autodesk Revit tools for semi-submersible wind turbine modelling.

Autodesk Revit’s Forms Tool is used to model the turbine’s primary components as separate Revit families and Void Forms Tool to create hollow geometries (see Fig. 3). Component materials shown in Appendix A are defined through Revit’s library according to offshore practices^18,30^.

Although multiple engineering standards for FWT exist (e.g. IEC 61400 series, ABS 195, DNVGL – ST – 0119)^31–33^, access barriers and licensing restrictions limit their direct application thus, data for component specifications are obtained from industry reports mentioned above.

### CE alignment and segmentation

**Fig. 4:**
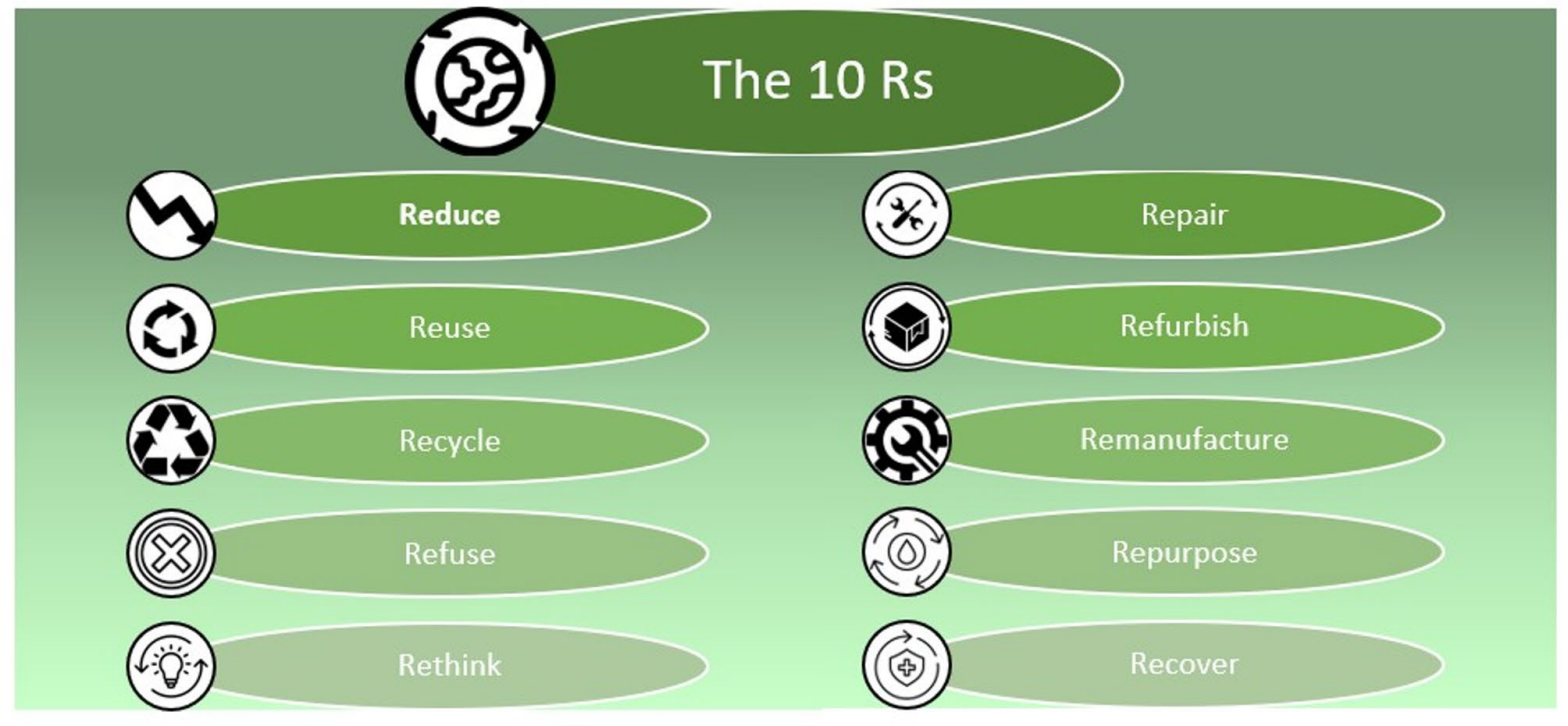
The 10Rs of CE.

**Fig. 5:**
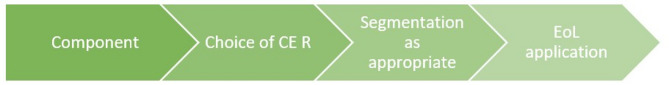
Component workflow to EoL stage.

The R-framework illustrated in Fig. 4, focusing on Reuse, Recycle, Repurpose, structures the classification of each component’s EoL pathway. Many CE principles apply primarily to design-stage interventions rather than decommissioning practice. The chosen Rs align directly with processes currently achievable at EoL. Within Revit, this framework is operationalised by modelling components as either whole units or subdivided elements depending on their circular pathway (see Fig. 5). For instance, items designated for reuse are retained as intact families, while those marked for recycling or repurposing are broken into subcomponents to represent realistic recovery routes. Embedding these Rs ensures material type, lifecycle phase and EoL pathway are consistently transferred throughout the workflow, creating a technical and conceptual link between design modelling and deconstruction planning.

### Phased deconstruction and export to Navisworks manage

**Fig. 6:**
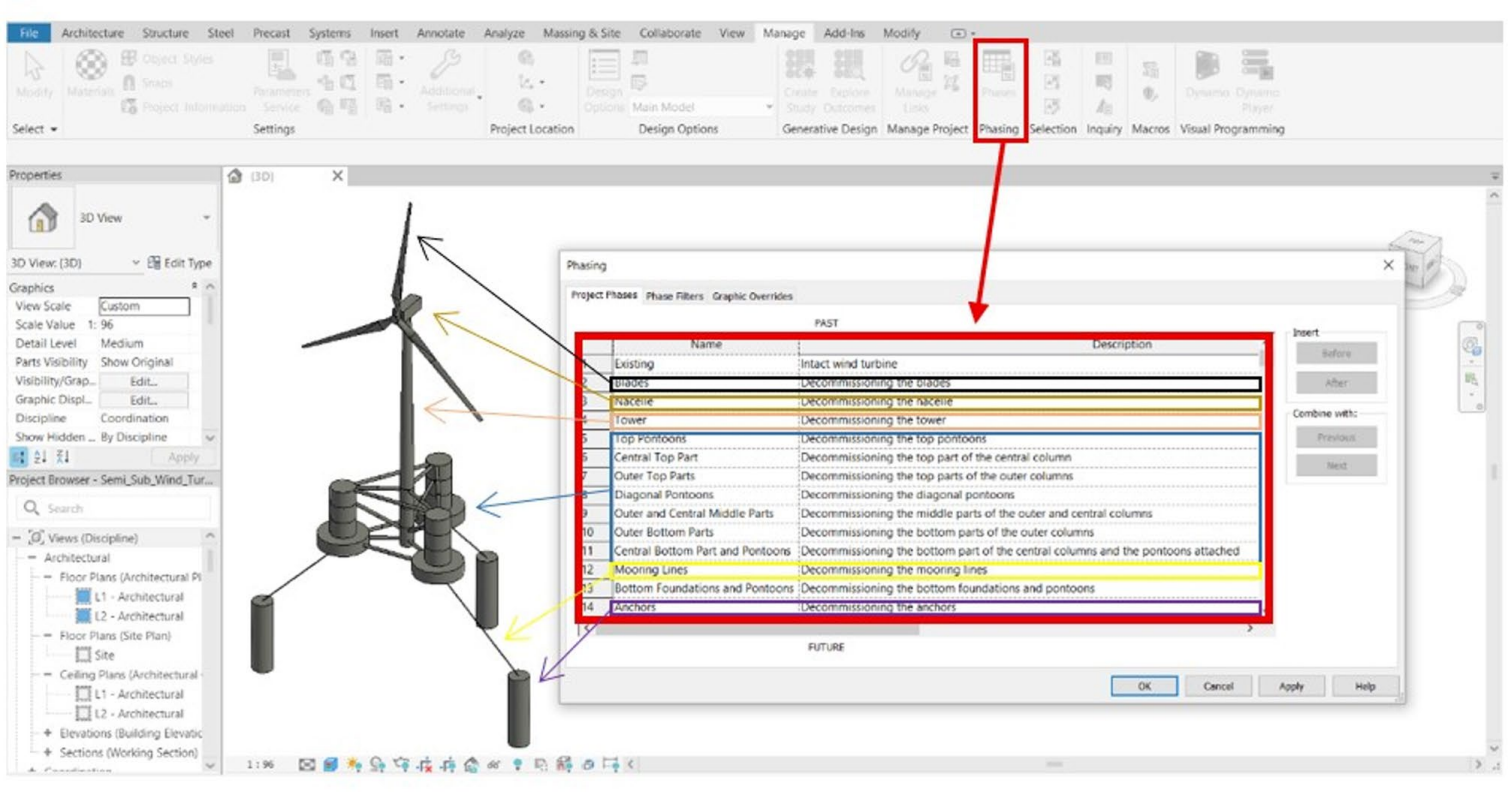
Phasing of decommissioning process in autodesk Revit.

**Fig. 7:**
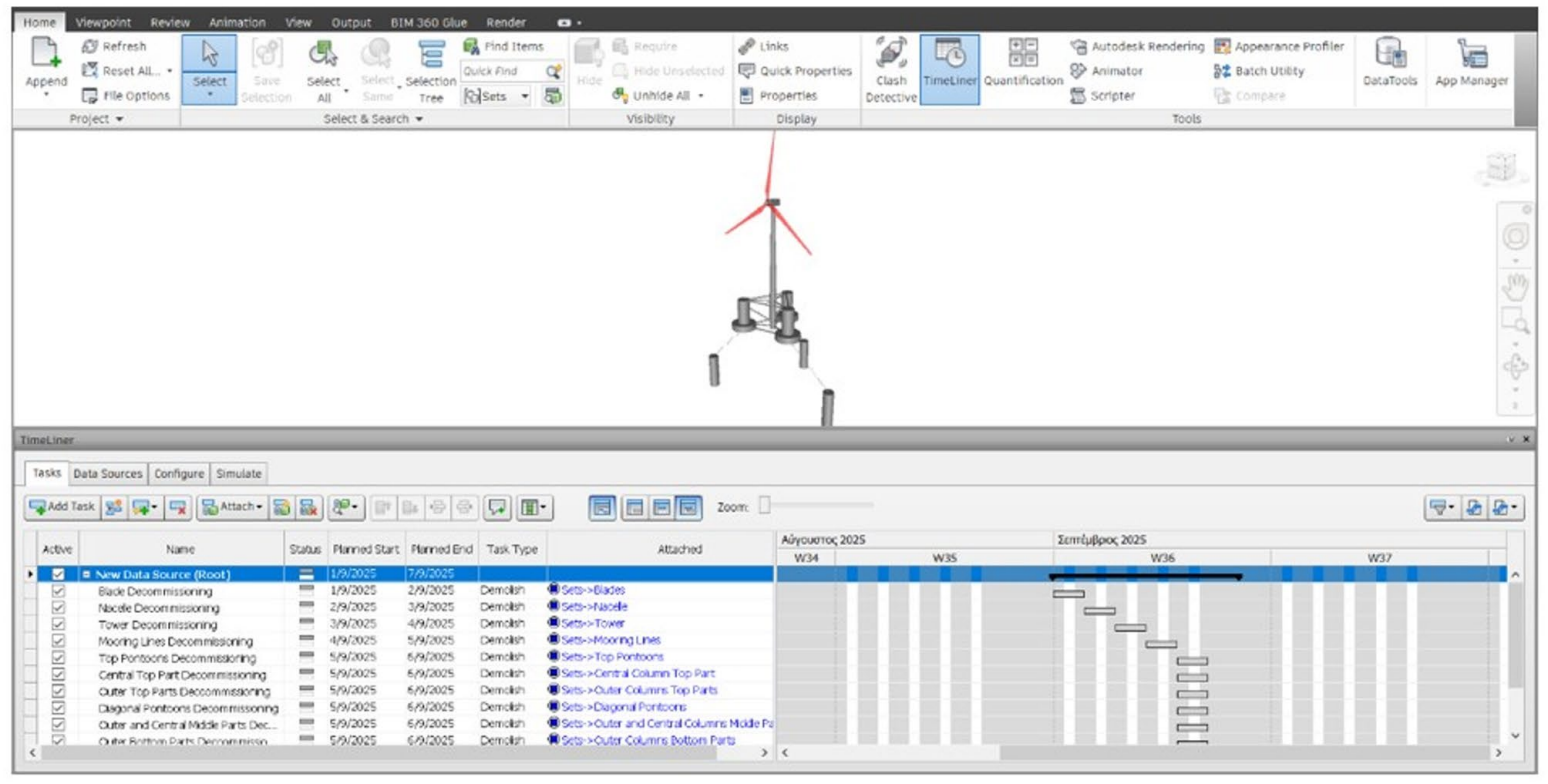
Implementation of BIM Navisworks to FWT.

The segmented Revit model is assigned dismantling phases using the Phases tool, with each component linked to its removal stage (see Fig. 6). The model is then exported in NWC format and imported into Navisworks Manage where the Timeliner tool is used to build a dismantling schedule that connects each element to a decommissioning activity and duration (see Fig. 7).

This mirrors offshore operational practice while allowing a clear visual simulation of the process. By embedding phased logic within the BIM workflow and transferring it into a time-based simulation, Navisworks supports optimised deconstruction planning, providing enhanced visualisation for more efficient scheduling and resource allocation^34^.

### Cost–time-CO₂ analysis

Cost, time and CO₂e emissions for each decommissioning phase are calculated by integrating BIM-derived mass data with secondary sources including peer-reviewed studies, industry reports and LCA. The Revit model provides component volumes which, when multiplied by material densities, yield the mass of each element. These values are then combined with unit rates for dismantling, operational costs and carbon emissions, allowing scenario-specific estimates of cost, duration and emissions.

The decision to rely on secondary data sources is justified by their credibility, coverage and replicability. Using these established references ensures consistency with recognised standards like ISO 14,044 for LCA, EN 15,804 for construction product declarations and PAS 2080 for carbon management in infrastructure^35–37^ and provides confidence that results are both scientifically reliable and directly comparable with wider literature. This approach avoids speculative assumptions and allows outputs to be grounded in recognised sector practice.

To ensure transparency and comparability with existing LCA studies, the carbon assessment in this research adopts clearly defined methodological assumptions, including the functional unit, system boundary, and allocation approach for recycled materials. The functional unit for the carbon assessment is defined as the end-of-life treatment of a FWT system, with component-level analysis conducted for major structural elements, including blades, nacelle, tower, floating platform, mooring lines, and suction anchors. Where applicable, results are also presented per component (e.g., three blades per turbine) to ensure consistency with existing literature and enable scenario comparison.

The carbon assessment adopts a cradle-to-end-of-life system boundary, focusing on the decommissioning and material recovery stages. The following processes are included:

- **Included Processes**: (1) Decommissioning and dismantling operations, (2) Cutting, separation, and pre-treatment processes, (3) Transportation to recycling, reuse, or disposal facilities, (4) Recycling, repurposing, and disposal processes and (5) Secondary material recovery and processing.
- **Excluded Processes**: (1) Original manufacturing and installation phases, (2) Operational and maintenance activities during service life, (3) Internal mechanical and electrical subsystems (due to minimal contribution to total material mass and waste streams).

This boundary definition aligns with the study’s objective of evaluating circular-economy performance in end-of-life management.

The three scenarios represent varying end-of-life (EoL) conditions of components, reflecting different levels of degradation and corresponding circular economy opportunities:

- *Scenario 1 – Optimal Condition*: Assumes near-perfect component condition at end-of-life, representing a best-case scenario in which components are suitable for direct reuse or repurposing with minimal refurbishment, thereby maximising material retention and circular value.
- *Scenario 2 – Moderate Degradation*: Introduces minor damage or wear, allowing for partial reuse of selected components. At the same time, other materials are directed towards downcycling or lower-value recycling pathways due to reduced performance or quality constraints.
- *Scenario 3 – Severe Deterioration*: Represents substantial degradation at end-of-life, limiting recovery options primarily to advanced recycling technologies or, where recovery is not technically or economically viable, final disposal.

**Fig. 8:**
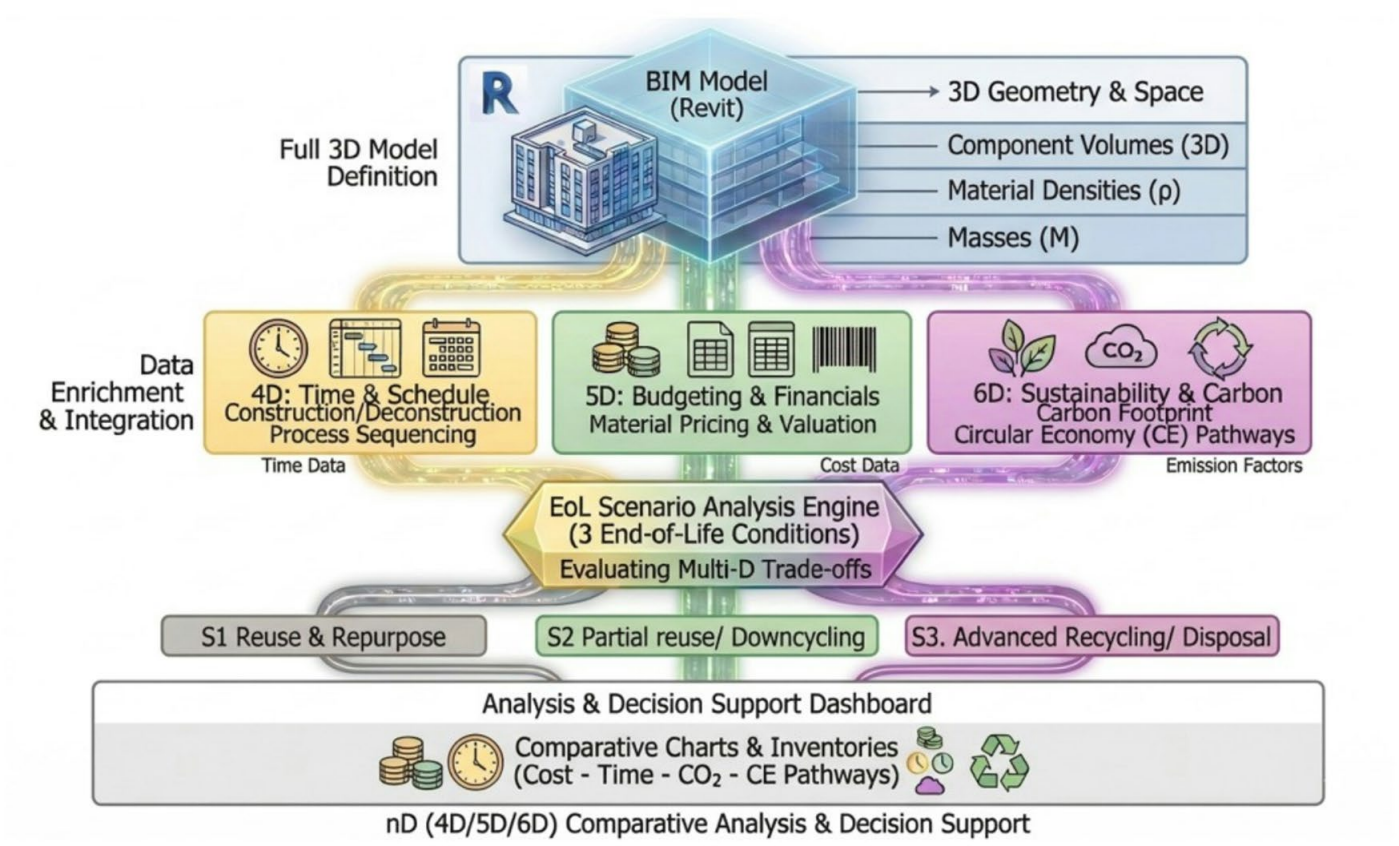
Analysis workflow.

The workflow (see Fig. 8) reflects the dimensional expansion of BIM, where geometric data (3D) underpin time (4D), cost (5D) and sustainability assessments (6D). All figures are compiled for three EoL scenarios and cross-checked against multiple references to ensure consistency. Outputs are visualised through comparative charts and linked to material inventories, ensuring alignment with CE pathways.

During the recycling and decommissioning phase, cost, duration, and carbon emissions constitute the primary indicators for evaluating sustainability performance. These metrics provide a comprehensive assessment of economic viability, operational efficiency, and environmental impact, which are critical considerations EoL management of complex infrastructure systems. However, these indicators are inherently dynamic and sensitive to multiple variables, including dismantling strategies, material recovery rates, transportation distances, logistical constraints, and the treatment methods adopted under different decommissioning scenarios. Consequently, evaluating sustainability performance requires a structured and systematic framework capable of capturing these variations and quantifying their impacts.

To address this challenge, the proposed framework quantifies three key performance metrics for each recycling and decommissioning scenario: total decommissioning cost (£), project duration (days), and lifecycle carbon emissions (tCO₂e). The total decommissioning cost includes expenses for dismantling operations, transportation, material processing, recycling, disposal, and associated labour and equipment requirements. Project duration reflects the time required to complete decommissioning activities, including mobilisation, dismantling, transportation, and treatment processes. Lifecycle carbon emissions account for greenhouse gas emissions generated throughout the decommissioning phase, including equipment operation, transportation activities, and material processing. The quantification of these indicators enables direct comparison between alternative recycling and decommissioning strategies and supports evidence-based, sustainability-driven decision-making.

The importance of these indicators is particularly pronounced in large-scale infrastructure projects, such as floating offshore wind turbines (FOWTs), where decommissioning activities involve substantial material volumes, complex logistics, and extensive processing requirements. In such contexts, even marginal improvements in cost efficiency, project duration, or carbon emissions can produce considerable cumulative benefits at the project or network level. These improvements may translate into significant economic savings, reduced environmental impacts, and enhanced operational efficiency across large-scale deployment scenarios.

BIM plays a critical role in managing these dynamic parameters by enabling integrated data management, real-time updates, and scenario-based analysis. BIM facilitates the organisation, visualisation, and analysis of large datasets, including material inventories, component specifications, transportation requirements, and recycling or disposal options. Through this capability, stakeholders can dynamically evaluate trade-offs between cost, time, and carbon emissions across multiple decommissioning scenarios. This integrated approach supports more efficient planning, improves transparency in decision-making, and enhances sustainability performance during the recycling and decommissioning phases.

The detailed design specifications of floating offshore wind turbine components—including blades, nacelle, tower, outer top, bottom, and central buoyant columns, pontoons, mooring lines, and suction anchors—are presented in Appendix A. Furthermore, the detailed calculations for each component under different conditions and decommissioning scenarios are provided in Appendix B. These appendices support the implementation of the proposed framework and ensure transparency and reproducibility of the analysis.

## Results

This section presents the outputs of the analysis, integrating BIM-derived mass data with cost, time and CO₂e performance metrics. Baseline masses are first calculated using Eq. 1^38^ and are detailed in Appendix A, combining volumetric outputs from the Revit model with material densities from literature^39^.

1 $$\:Mass\:\left(tn\right)=Volume\:\left({m}^{3}\right)\times\:Density\:\left({kg}\left/\:{{m}^{3}}\right.\right)$$

Results are structured around three EoL scenarios representing progressive levels of deterioration. *Scenario 1* assumes near-perfect component condition and reflects best-case outcomes for reuse and repurpose. *Scenario 2* introduces minor damages, allowing partial reuse or downcycling through lower-value recycling. *Scenario 3* represents severe deterioration at EoL, limiting options to advanced recycling, or disposal. These scenarios are selected to mirror real-world uncertainties in offshore environments. Their inclusion provides a structured framework to test how condition affects recovery potential, resource efficiency and CE outcomes. In practice, turbines rarely exit service in uniformly good condition^6^. By comparing three realistic pathways, the analysis captures the operational variability that supports both technical feasibility and sustainability performance.

Aggregate outputs for cost, time and CO₂e are presented in tables and visualisations, providing a reproducible framework for evaluating CE opportunities and decommissioning strategies in future offshore wind projects. Full breakdowns of component-specific results are presented in Appendix B.

### Offshore logistics

**Fig. 9:**
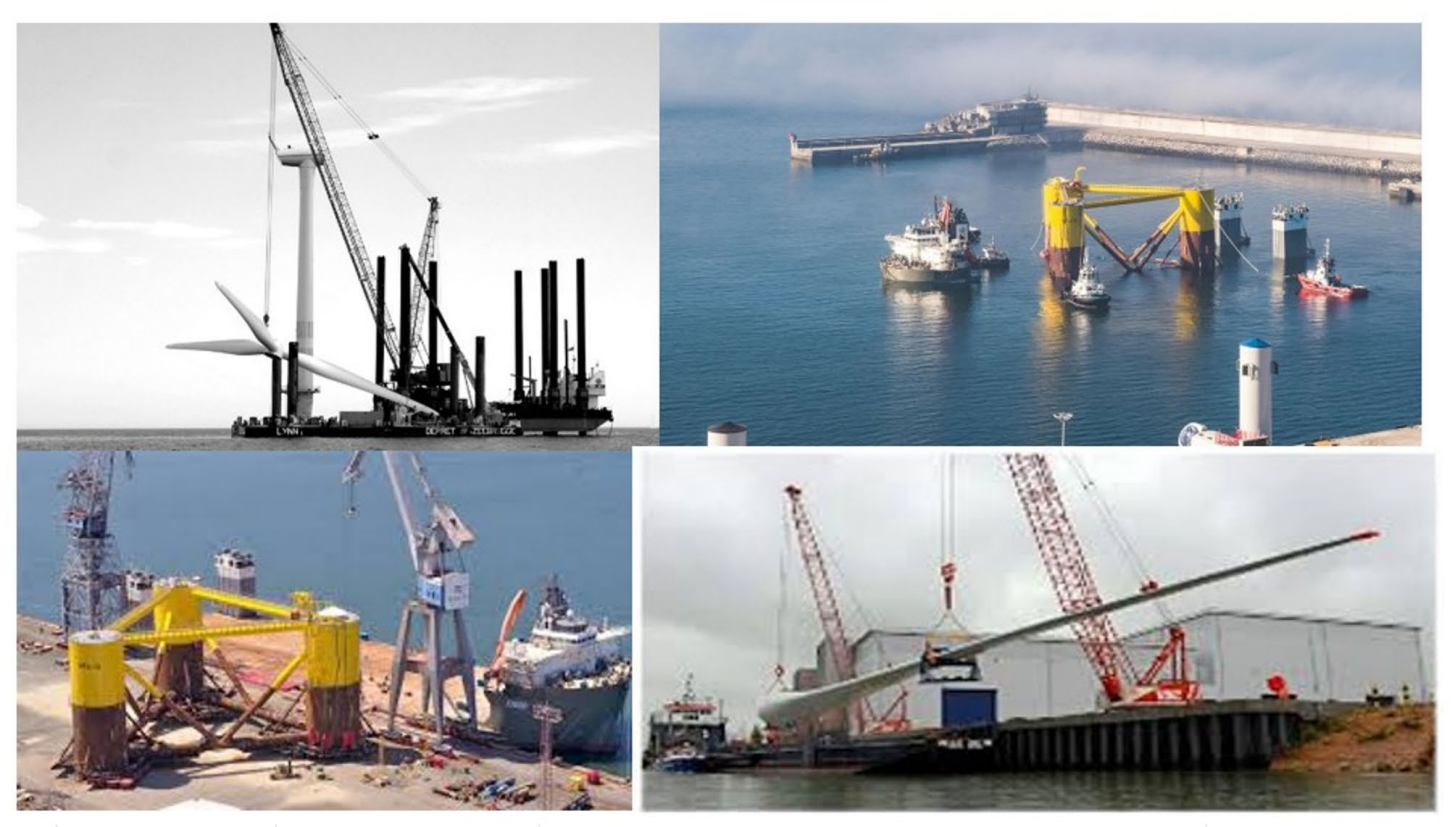
Operations of vessels^42–44^.

Marine logistics reinforce every phase of offshore decommissioning and represent a dominant driver of cost, duration and carbon emissions. In this analysis, three Jack-Up Barges (JUBs) and six tugboats are mobilised to dismantle, tow, transport and offload components (see Fig. 9)^40^, with an average port-to-site distance of 20 km^41^.

**Fig. 10:**
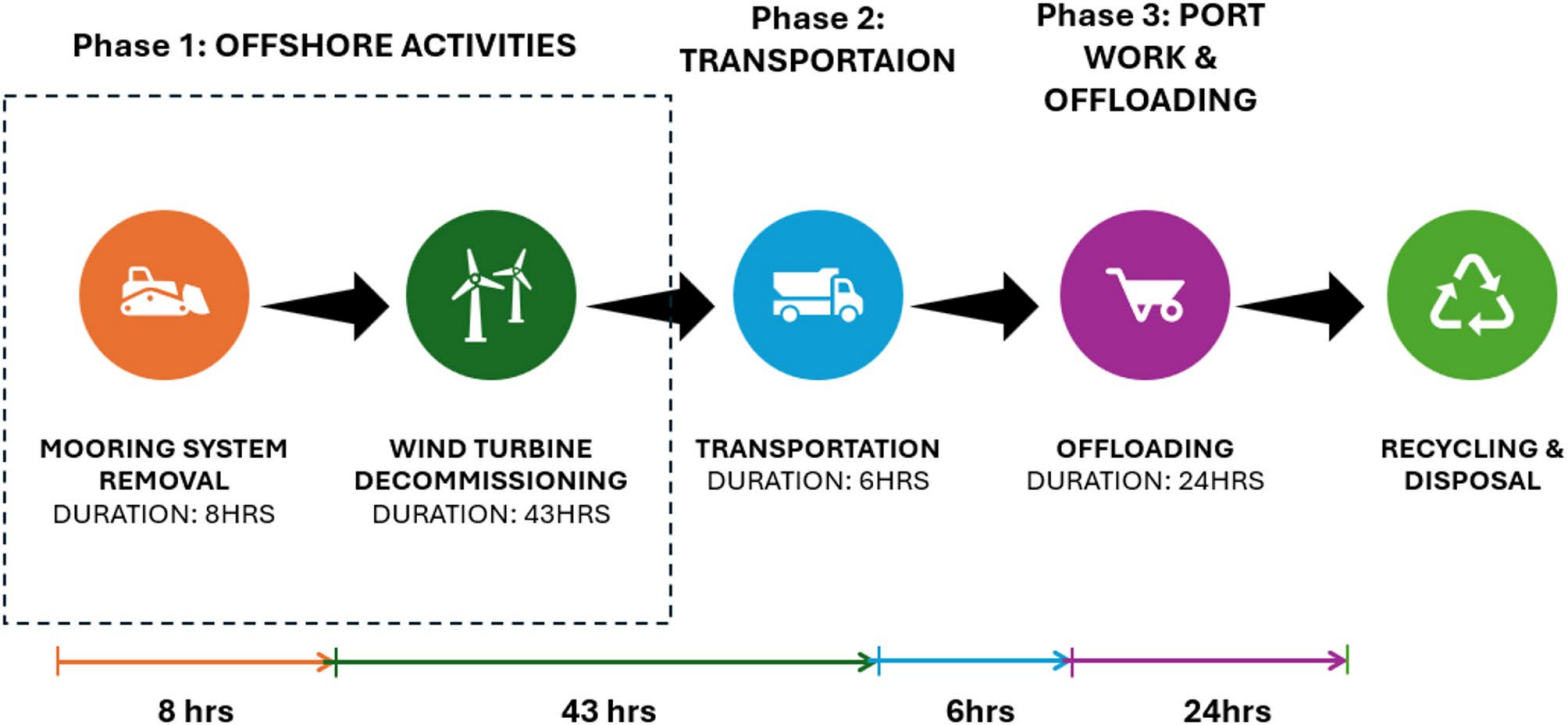
Duration of marine logistics activities.

The total cost of marine operations is calculated at £633,000, comprising vessel charter fees (£576,000) and tugboat support (£60,000)^40^. Over an estimated four-day operational window, the following tasks presented graphically in Fig. 10 are completed:

- 43 h for turbine component lifts^45^,
- 8 h for removal of mooring system^46^,
- 6 h estimated for transportation and.
- 24 h for offloading.

**Fig. 11:**
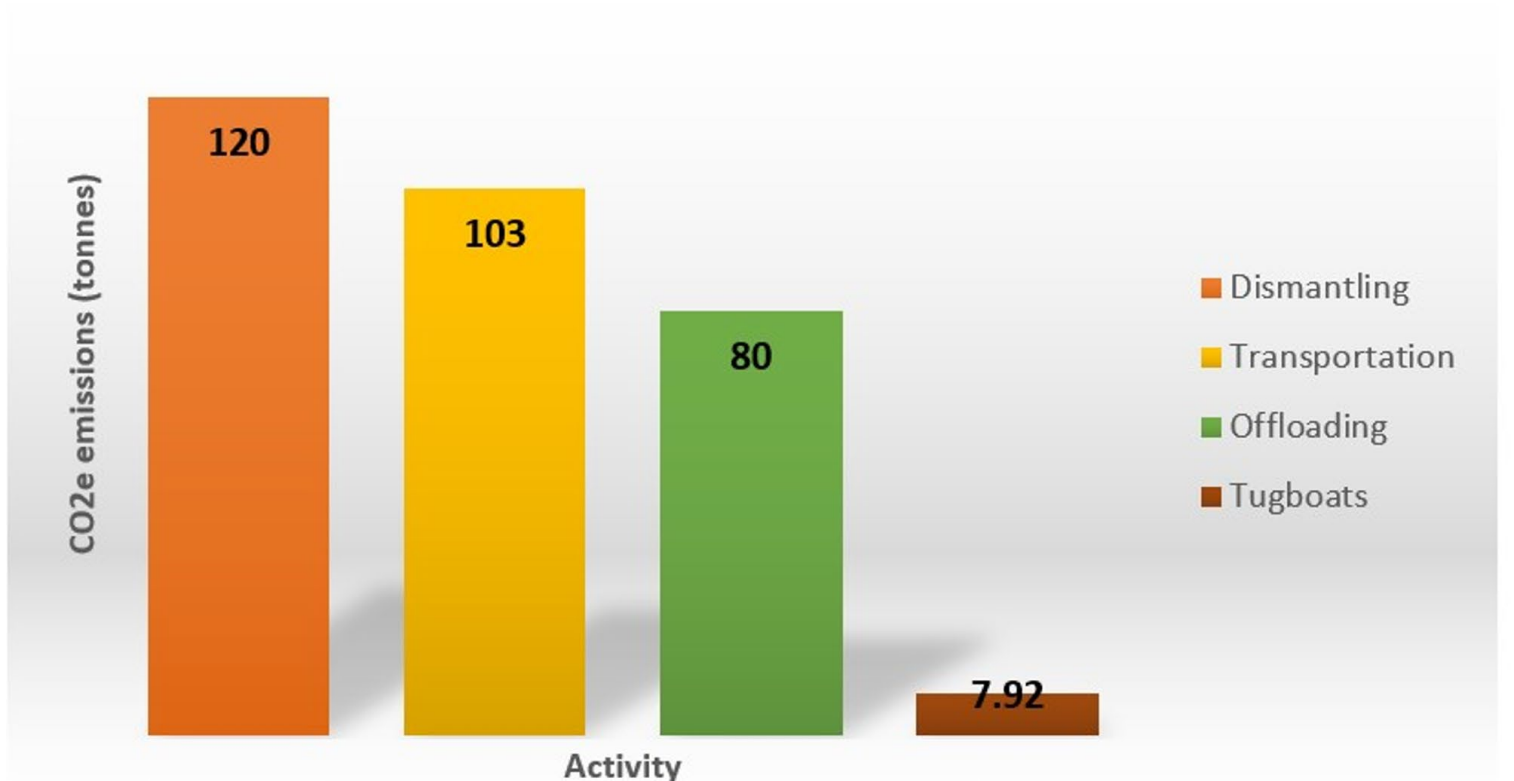
Carbon emissions of marine logistics activities.

Environmental performance substantiates the significance of logistics. The four-day campaign emits 302.6 tonnes of CO₂e from JUB operations, with an additional 7.9 tonnes from tugboats. Most emissions stem from dismantling and transport activities^46–48^. These results, shown in Fig. 11, highlight that analysing logistics alongside component-level recovery scenarios could represent an essential lever for reducing offshore wind’s EoL footprint.

### Blades

**Fig. 12:**
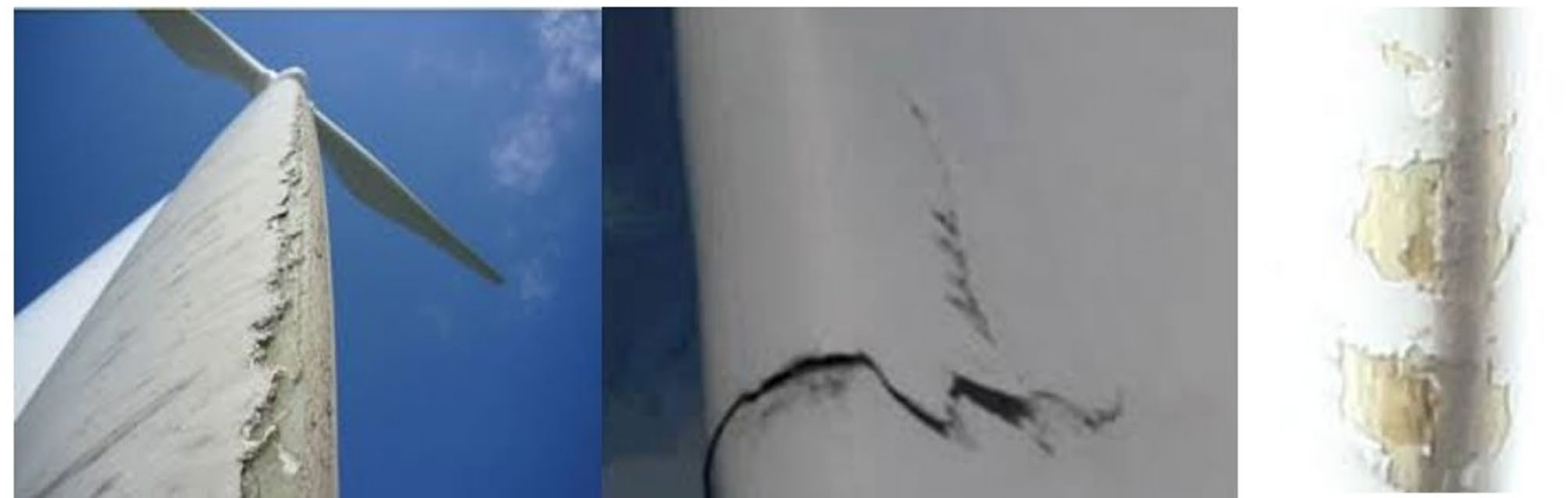
Erosion (**a**), cracking (**b**) and surface peeling (**c**) of wind turbine blade^49–51^.

Wind turbine blades represent one of the most challenging elements to process at EoL due to their Glass-Fiber Reinforced Plastic (GFRP) structure. Prolonged exposure to high wind loads, UV radiation and marine moisture can result in common blade degradation including leading-edge erosion, matrix cracking and surface peeling (see Fig. 12a–c).

**Fig. 13:**
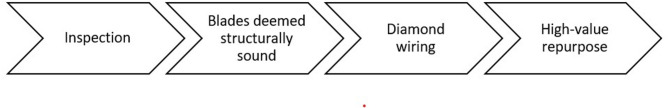
Scenario 1 workflow.

**Fig. 14:**
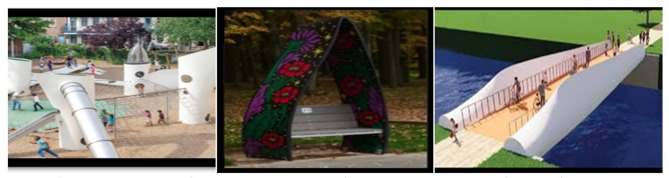
Repurposing blades after EoL^5,25^.

For *scenario 1* (as displayed in Fig. 13), blades are assumed to be largely intact, allowing for direct repurpose after industry-standard inspections such as visual surveys, radiography, leak testing and ultrasonic NDT^52^. Sections are cut using diamond saws, with a processing duration of approximately 72 h for three blades^53^. The associated cost is around £10,000 and emissions reached 10 tonnes CO₂e^54,55^. This pathway aligns strongly with CE principles, as intact blade sections can be reused as playground structures, pedestrian bridges, benches etc. shown in Fig. 14, thereby extending material life with minimal processing.

**Fig. 15:**
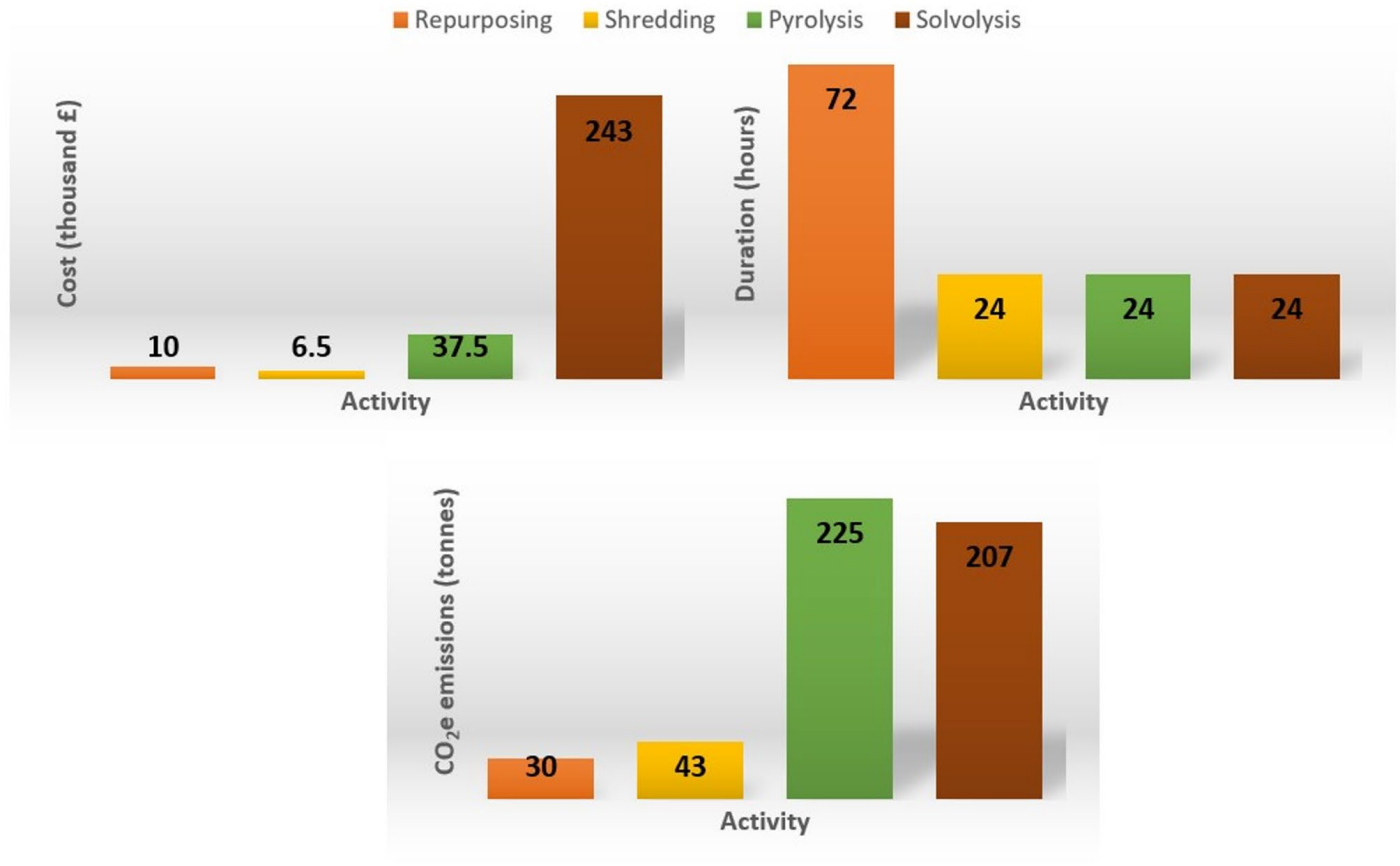
Cost, time and carbon data for blade processing.

*Scenario 2* targets blades with minor damage necessitating mechanical recycling through shredding. Mechanical repairing cost is estimated at £6,500, with insignificant duration and emissions of 43 tonnes CO₂e^55,56^. Recovered fibres and fillers can be used as additives in concrete or cement, supporting a scalable recycling route^57^.

In *scenario 3*, blades are subjected to heavy degradation and are suitable only for recycling. Thermal pyrolysis may require approximately one day per blade at a cost of £37,500 and 225 tonnes CO₂e emissions, while chemical solvolysis is significantly more expensive at £243,000 with 207 tonnes CO₂e^58–61^. Both techniques offer the potential to recover fibres for reuse in new composite products. Graphs in Fig. 15 present the calculated outputs.

Overall, the results demonstrate clear trade-offs and uncertainties, as described in Table 3 considering insights from Tao et al.^57^, ACP Wind Blade Working Group^56^ and Science for Environment Policy^61^.

**Table 3:** Pros, cons and uncertainties of blade recycling processes.

Recycling process	Pros	Cons	Uncertainties	References
Mechanical recycling (Shredding)	Simple process, low investment requirements	Limited material recovery, degradation of mechanical properties of recovered fibres	Restricted reusability due to fibre degradation	Tao et al.^57^
Thermal recycling (Pyrolysis)	High-resale value of recovered composites with high strength	Expensive, risk of excessive damage due to exposure to high heat	Uncertain process scale up	ACP Wind Blade Working Group and Science^56^
Chemical recycling (Solvolysis)	Recovery of high-quality fibre and resin	Very expensive, technologically immature	Limited industrial deployment	Environment Policy^61^

### Nacelle

**Fig. 16:**
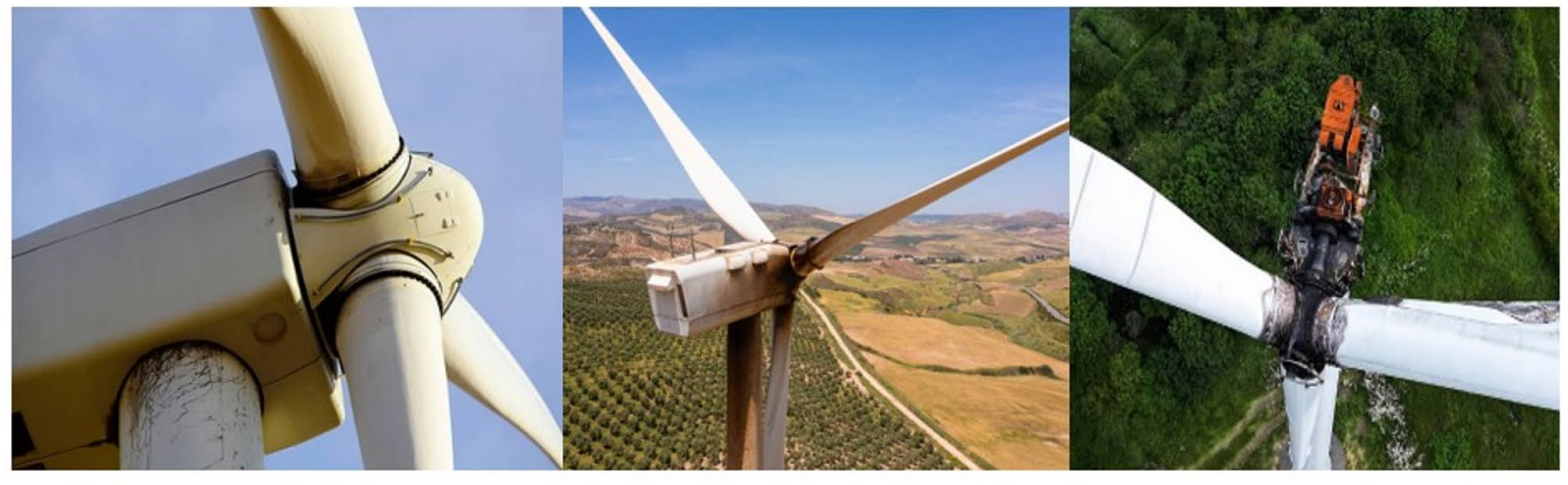
Surface wear (**a**, **b**), compromised shell due to weather (**c**)^28^.

The nacelle shell, built primarily from GFRP^28^, is prone to surface wear and storm-related damage at EoL as shown in Fig. 16.

In Scenarios 1–2, where the structure remains intact or repairable, it can be repurposed at negligible cost, time and emissions. Example applications include shelters, storage units, or marine enclosures. This extends lifecycle value without energy-intensive processing and represents the most CE-aligned pathway.

In *Scenario 3*, recycling is necessary. Mechanical shredding is proved most efficient (£11,600, < 1 day, 77 tCO₂e) but produces only low-grade fillers. Pyrolysis (£68,300, 6 days, 413 tCO₂e) offers partial fibre recovery, while solvolysis achieves higher fibre quality (£445,500, 380 tCO₂e) but at prohibitive cost and scale^56^.

**Fig. 17:**
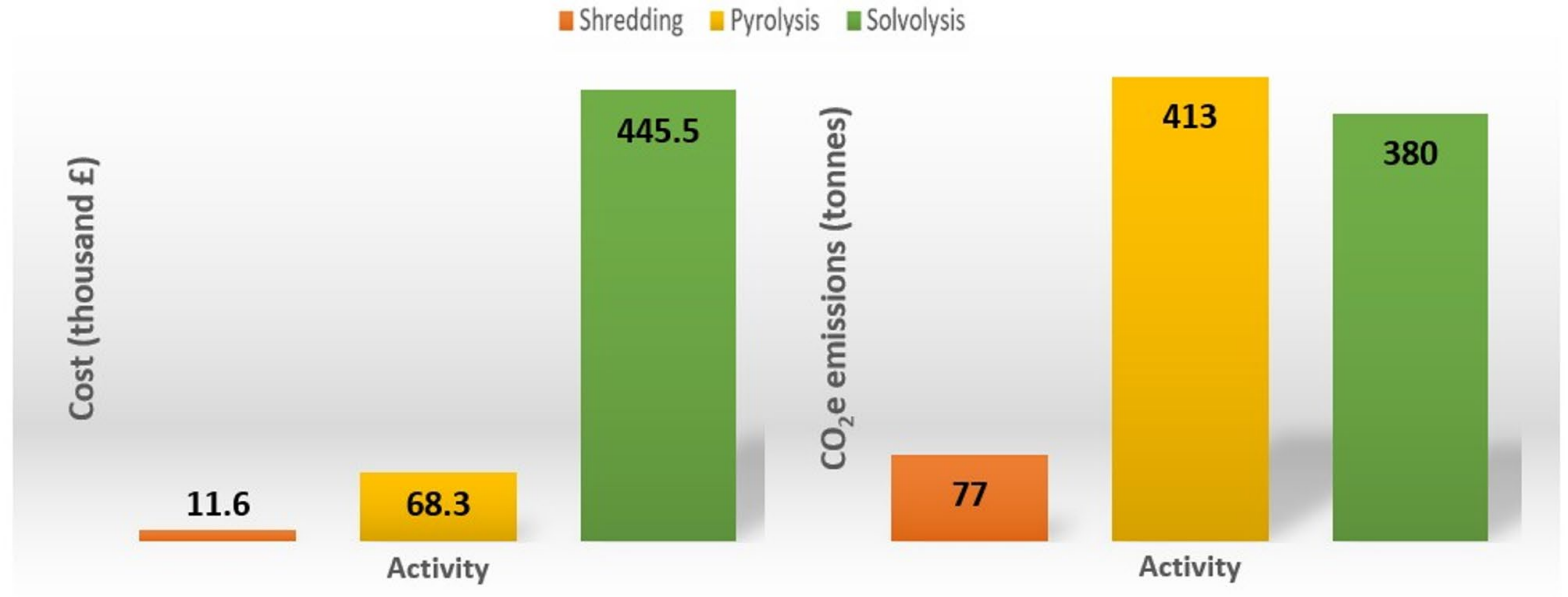
Cost and carbon data for nacelle recycling.

Figure 17 compares these pathways, with detailed values in Appendix B. The results confirm that reuse/repair pathways clearly outperform recycling in both cost and emissions, while recycling involves trade-offs between efficiency and fibre quality (see Table 1).

### Tower

**Fig. 18:**
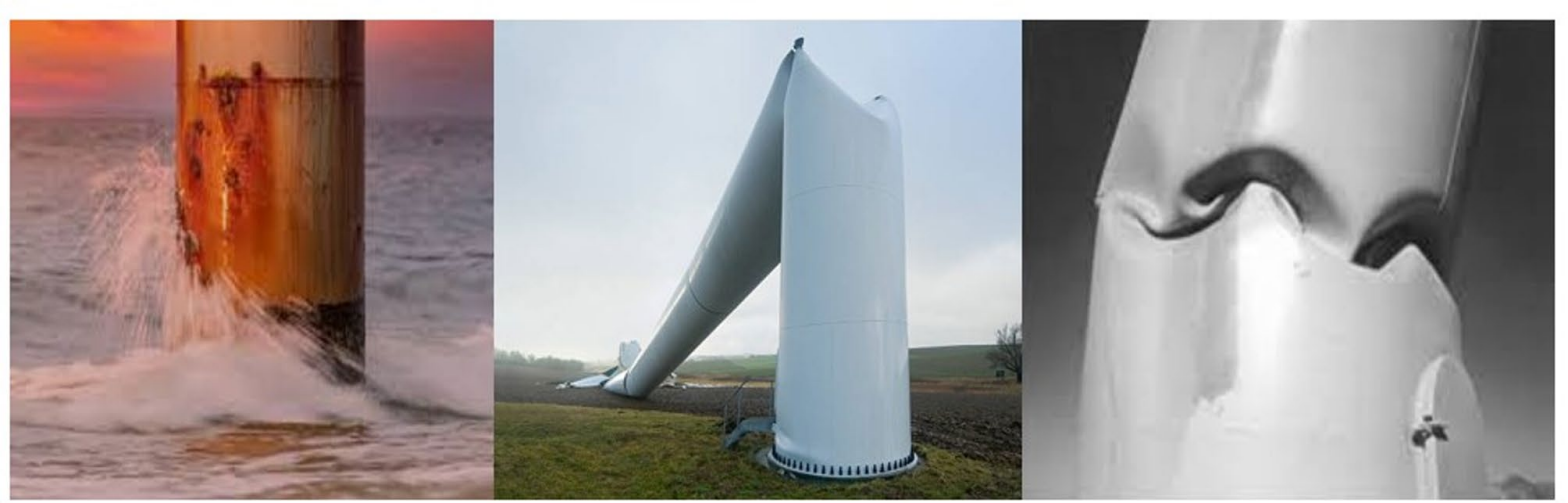
Corrosion (**a**), failure due to fatigue (**b**), severe deformation (**c**) of wind turbine tower^62,63^.

The tower, constructed with steel S355^18^ is vulnerable to corrosion, structural fatigue, severe deformation etc. as seen in Fig. 18.

**Fig. 19:**
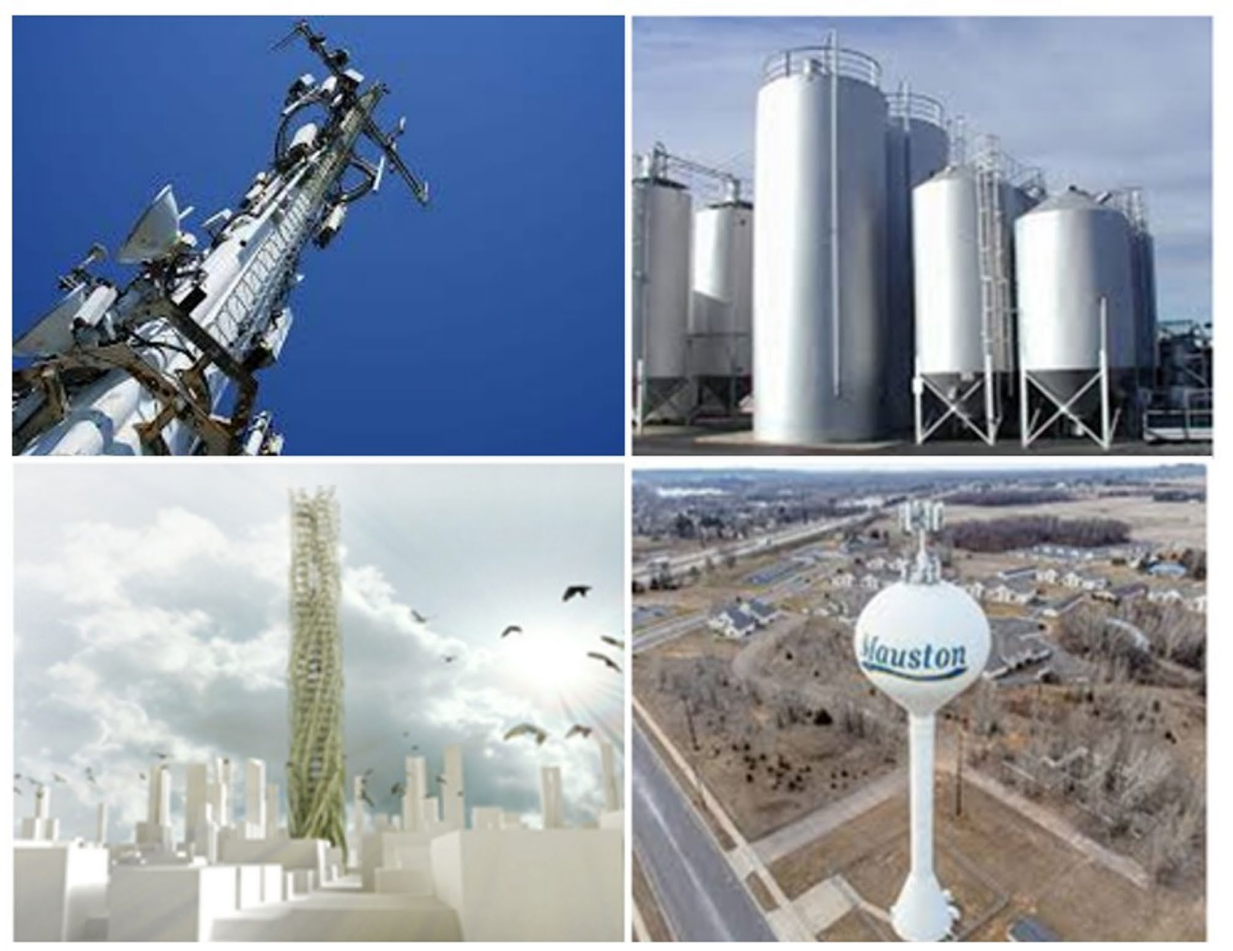
Repurposing wind turbine after EoL^66,67^.

*Scenarios 1–2* assume the tower is intact or affected only by minor deterioration. Industry-standard inspections—including ultrasonic weld testing, magnetic particle inspection and corrosion mapping—can be used to assess structural integrity of S355. If damage is limited, reuse or repurposing remains viable: applications include telecom masts, water towers, silos, or vertical farming structures (see Fig. 19). Repair costs range from £14,000 to £93,000 with negligible added emissions^64,65^. This pathway supports CE by extending structural lifecycle and displacing demand for virgin steel.

*Scenario 3* considers extensive degradation that prevents reuse with recycling becomes the only viable route. Recovery via Electric Arc Furnace (EAF) yields lower emissions (78.5 tCO₂e) compared to the Blast Furnace–Basic Oxygen Furnace (BF-BOF) process (437 tCO₂e)^68^. Recycling costs, including dismantling, are £40,000^69^. Despite its efficiency, recycling and processing S355 poses uncertainties (see Table 4)^68–70^.

**Table 4:** Pros, cons and uncertainties of processing S355.

Process	Description	Pros	Cons	Uncertainties	References
Welding S355	Melting process	Strong, durable, cost-effective results	Heat-Affected Zones (HAZ)	Possible compromise of long-term performance	Eurocode 3: Design of steel structure – Part 1–8: Design of joints^71^
EAF	Use of 105% scrap steel	Use of scrap steel only with minimal loss	Lack of scrap steel worldwide	Minimal scalability	JRC141817^72^
BF-BOF	Use of 14% scrap steel	Easier application	Production of large CO2 amounts	Conflicting with decarbonization goals	World Steel Association^73^

Overall, reuse/repair offers the highest CE value when inspections confirm integrity, while recycling performance depends on future access to low-carbon steelmaking routes.

### Platform

**Fig. 20:**
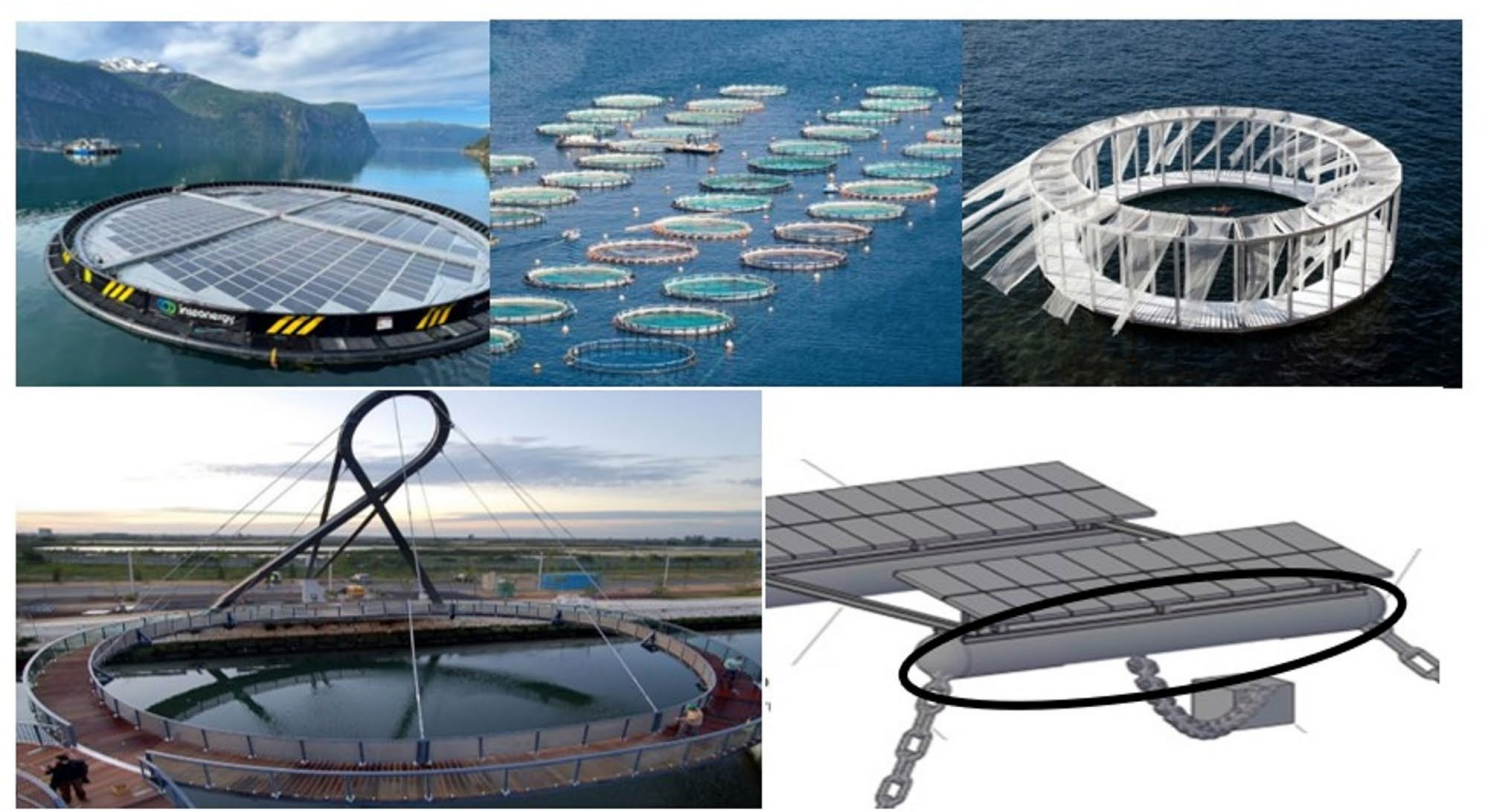
Repurposing platform components after EoL^75–77^.

The floating platform, consisting of buoyant columns and interconnecting pontoons, is manufactured from stainless steel (SS) 316 L. Typical EoL issues include surface cracks, pitting, fatigue and corrosion. *Scenario 1* assumes that inspection reports—covering ultrasonic weld testing, corrosion mapping and visual surveys—confirm structural integrity. In such cases, elements can be directly repurposed with columns as aquaculture cages, floating solar supports or offshore platforms and pontoons for marine infrastructure (see Fig. 20). Plasma cutting enables segmentation, producing no direct CO₂e but generating hazardous particulates^74^.

Repairing in *scenario 2* allows reuse with minimal carbon impact (< 1 kg CO₂e from welding), though costs increase to £66,000 – £212,500 depending on intervention^65,78^. This extends the service life of high-grade steel with relatively low emissions, reinforcing CE value.

In *scenario 3* where severe degradation occurs, recycling becomes the only option. Melting the platform’s SS 316 L recovers material but releases 431.5 tCO₂e^79^. While material value is retained, the process is carbon-intensive and less aligned with CE objectives.

Overall, the platform illustrates a tiered EoL pathway: inspection-led direct reuse, repair-enabled repurposing and recycling only as a last resort. However, implications of SS 316 L processing presented in Table 5^80–82^, should be considered early on the project to ensure optimised treatment.

**Table 5:** Pros and cons of processing SS 316 L.

Process	Pros	Cons
Plasma cutting	Precise cuts, maximisation of material utilization	Expensive, energy-intensive
Recycling 316 L	Preserved physical and chemical properties, durable outcome, maintained value	High CO_2_ emissions, energy-intensive

Detailed segmentation data about cost and time estimations regarding component cutting and related processes is not typically found in public-facing academic or government offshore wind cost reports, especially for semi-submersible wind turbines.

### Mooring lines

**Fig. 21:**
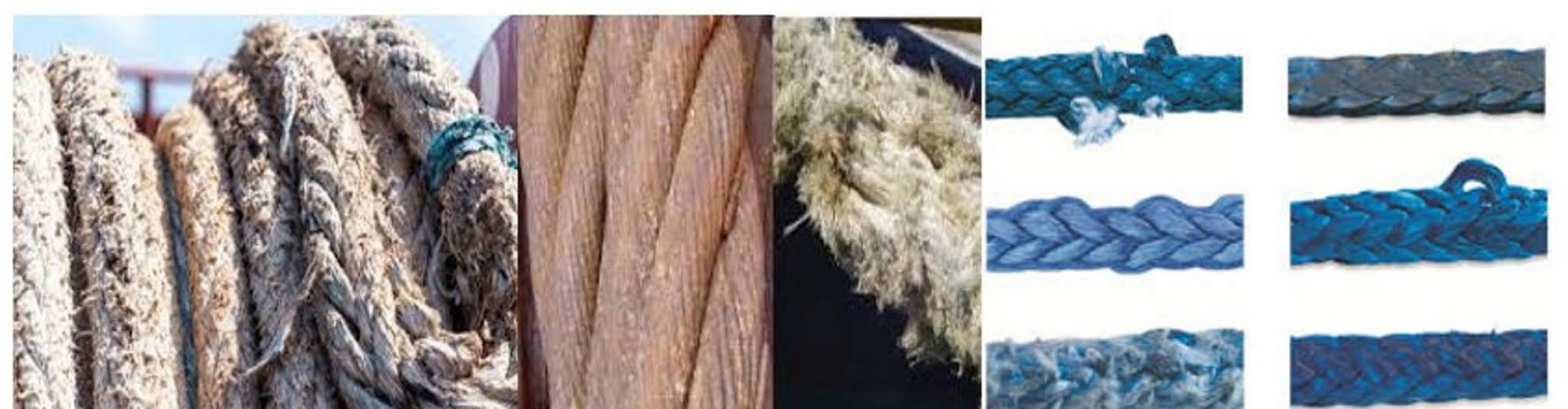
Damaged mooring lines^83^.

The floating platform is secured through polyester taut mooring lines connected to suction anchors^30^. Over their service life in harsh marine environments, they are susceptible to UV degradation, fraying and fatigue-related fractures as presented in Fig. 21.

**Fig. 22:**
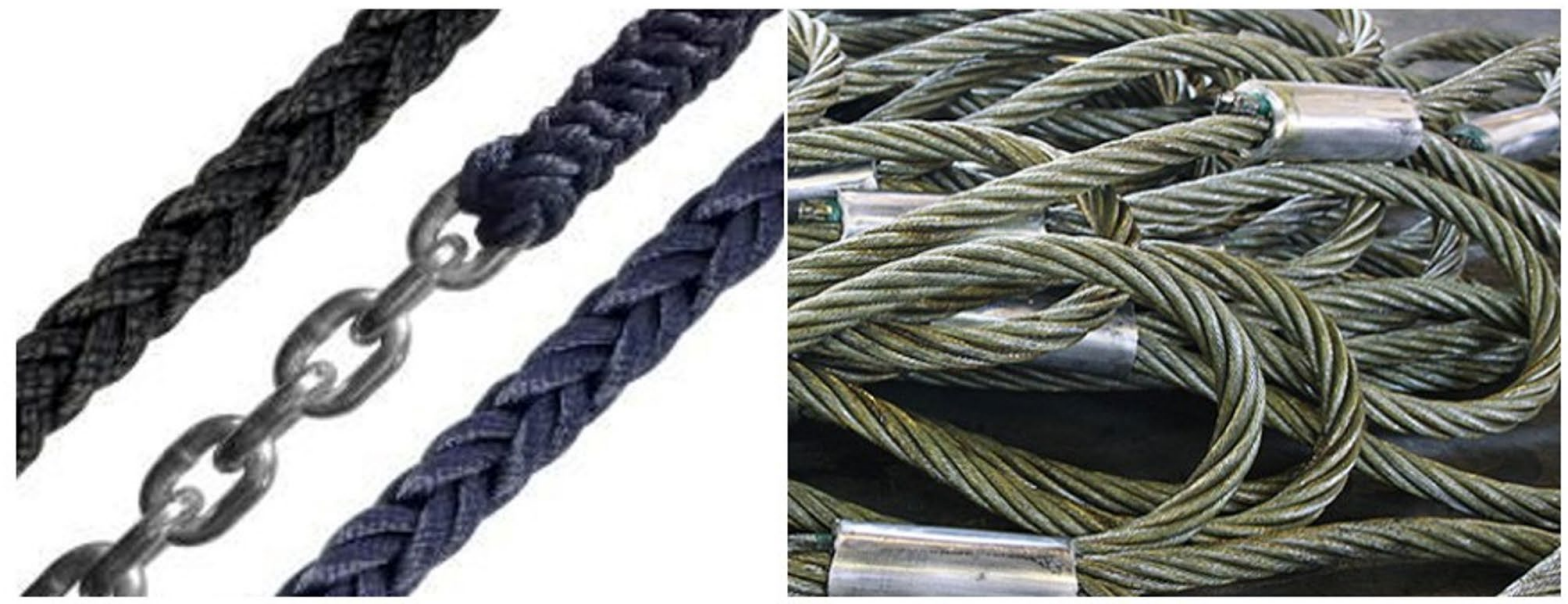
Reuse and repurpose of mooring lines after EoL^84^.

In *scenario 1*, lines can be inspected and directly reused in future offshore or marine projects, e.g. as anchor slings or primary mooring ropes (see Fig. 22). *Scenario 2* allows downcycling through segmentation and re-splicing for use as secondary ropes in less strength-intensive applications. These two cases present cost-efficient, low-carbon pathways as they extend material lifetimes within the CE.

In contrast, reuse in *scenario 3* is unfeasible and recycling is still technologically immature and commercially unavailable^85^. Consequently, the most realistic outcome is controlled disposal with limited resource recovery, carrying higher cost and emissions.

Although detailed public datasets on rope recycling are limited, the results confirm that *Scenarios 1–2* clearly outperform *Scenario 3*, demonstrating the importance of inspection-driven pathways that maximise reuse before considering disposal.

### Suction anchors

**Fig. 23:**
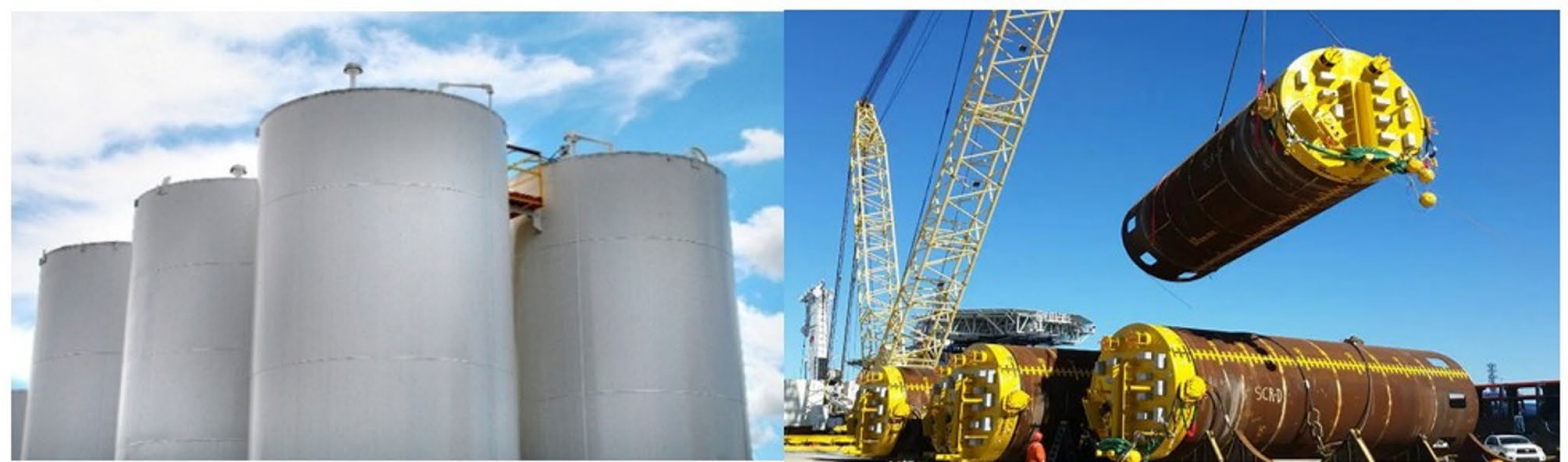
Reuse and repurpose of suction anchors after EoL^86^.

The three suction anchors, made of S355 steel^18^, are subject to long-term seabed exposure leading to corrosion, fatigue cracking and weld degradation, which underpin the three EoL scenarios. In *scenario 1*, anchors in near-perfect condition can be directly reused as test anchors or storage tanks (see Fig. 23) following mechanical and visual inspections. Similarly, in *scenario 2*, localised repairs and surface treatments enable repurposing, along with additional costs calculated at £15,000–£101,000^65^. Both scenarios reflect reuse as a higher-value recovery route.

Conversely, *scenario 3* considers recycling as the only viable pathway, with two processing options: EAF or BF-BOF resulting in 118 and 656 tCO₂e respectively^69^.

The results confirm that reuse and repair present the most circular options, while recycling through EAF provides the lowest-carbon fallback solution, aligning with CE goals of reducing virgin steel demand and landfill disposal.

### Navisworks implementation

Finally, with the help of Navisworks, time sequencing of the decommissioning phases is introduced. By assigning durations to each dismantling step, the FWT generates a sequential schedule that clearly illustrates how the decommissioning campaign progresses. This temporal profile allows identification of the project’s critical path, the distribution of time across phases and the dependencies between operations.

**Fig. 24:**
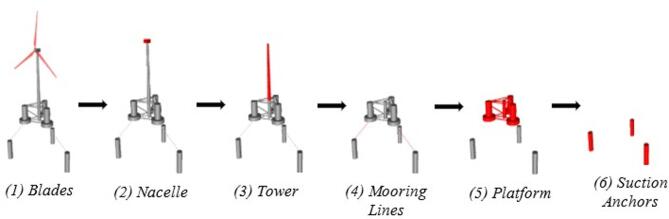
Navisworks sequence of wind turbine dismantling.

**Fig. 25:**
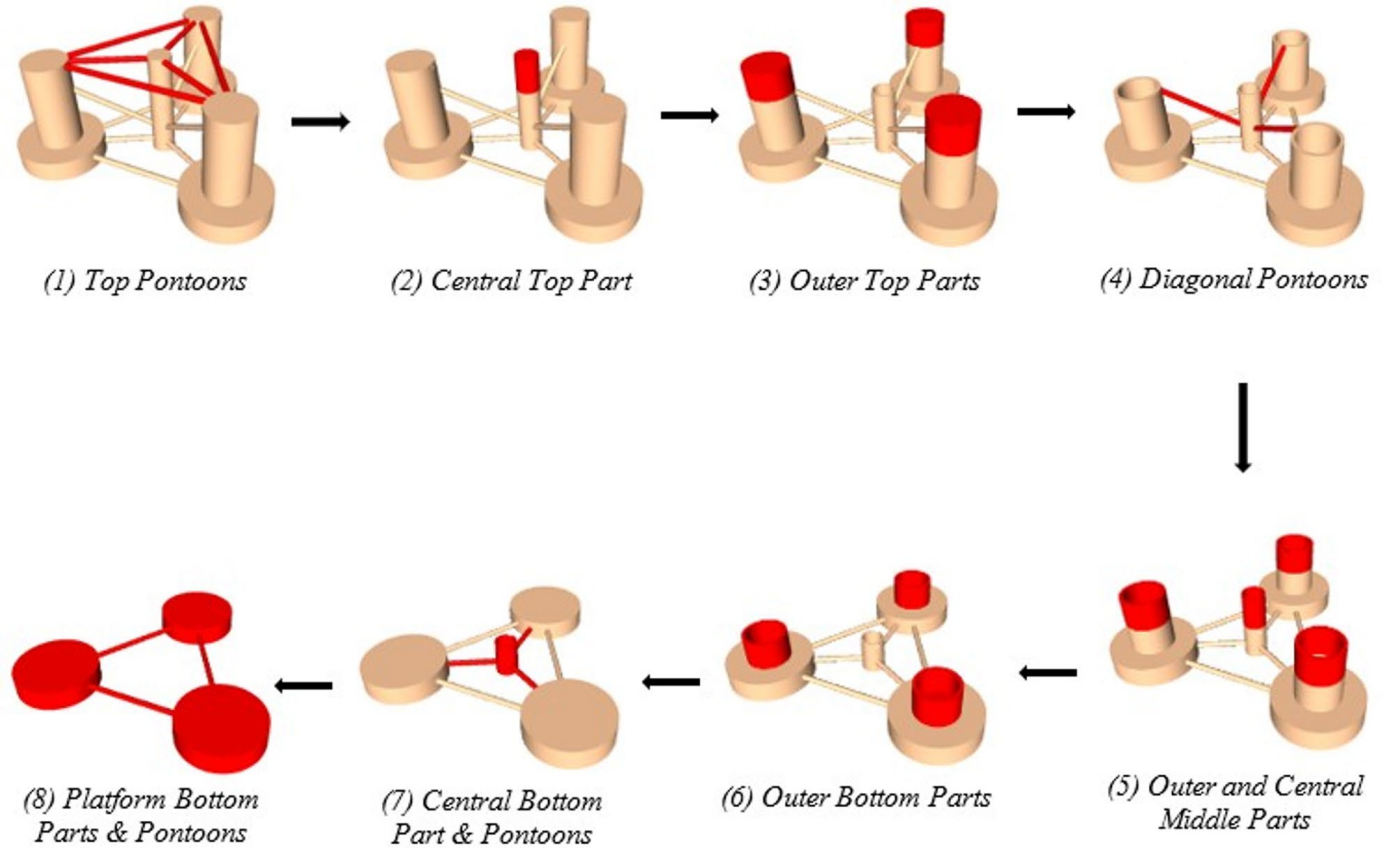
Navisworks platform segmentation sequence.

While the model also provides a visual animation of dismantling (see Figs. 24 and 25), its principal value lies in the structured breakdown of the turbine into components. By defining when and how each element is removed, Navisworks clarifies the dismantling workflow and prepares components for subsequent evaluation. In this way, time sequencing not only demonstrates the feasibility of the proposed method but also provides the foundation for integrating CE objectives once the parts reach EoL.

Marine logistics alone account for approximately £633,000, four operational days, and 310.5 tonnes CO₂e, demonstrating that offshore logistics represent a dominant contributor to decommissioning impacts. Component-level analysis further reveals substantial variation across scenarios. For example, blade repurposing in Scenario 1 results in relatively low impacts (£10,000, 72 h, 30 tCO₂e), whereas advanced recycling techniques such as solvolysis in Scenario 3 increase costs significantly to £243,000 and emissions to approximately 207–225 tCO₂e. Similarly, recycling of large steel components shows considerable environmental impacts, including 78.5–437 tCO₂e for tower recycling and 431.5 tCO₂e for platform recycling. These results highlight that reuse and repair pathways consistently cost less and emit less carbon than recycling options (Table 6).

Overall, the quantified results confirm that inspection-led reuse and repurposing strategies offer the most cost-effective and low-carbon pathways. At the same time, recycling serves as a necessary fallback when structural degradation prevents reuse.

**Table 6:** Comparative summary of cost, duration and carbon emissions for key end-of-life strategies.

Component/activity	Scenario	Cost (£)	Duration	CO₂e (t)	Key insight
Marine logistics	Decommissioning	633,000	4 days	310.5	Dominant contributor to overall impacts
Blades	Scenario 1 (Repurpose)	10,000	72 h	30	Lowest cost and emissions
Blades	Scenario 3 (Solvolysis)	243,000	~ 1 day/blade	207–225	Highest cost recycling option
Tower	Scenario 3 (EAF–BF/BOF)	~ 40,000	< 1 day	78.5–437	Large variation depending on process
Platform	Scenario 3 (Recycling SS 316 L)	—	—	431.5	High emissions due to steel processing

**Fig. 26:**
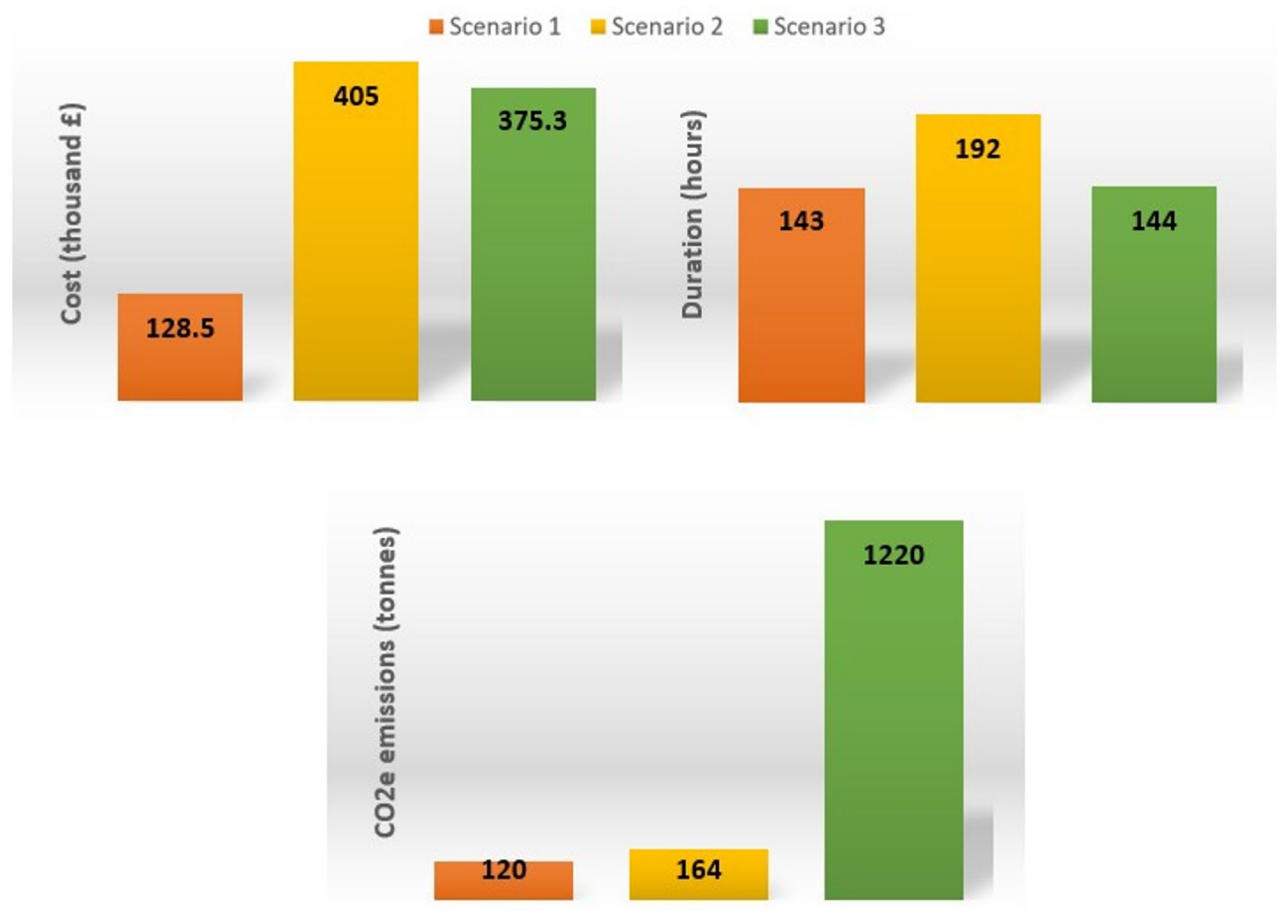
Total cost, time and carbon data.

Across all components, the results in Fig. 26 show that *Scenario 1* delivers superior performance, with lowest cost, time and carbon values while preserving maximum material utility. *Scenario 2* offers moderate circular value but introduces additional processing. *Scenario 3* is most emission-intensive, highlighting the need to avoid designs that default to recycling-only strategies. These contrasts illustrate why condition-based decommissioning frameworks are essential for aligning offshore wind with CE principles.

## Discussion and limitations

The analysis demonstrates that the three EoL scenarios provide a structured and realistic way to explore how conditions at decommissioning influences circular outcomes. Their rationale stems from operational variability in offshore environments where components may remain intact, incur moderate deterioration, or sustain severe. *Scenario 1* reflects the best-case pathway where inspections confirm integrity, allowing direct reuse and repurpose. *Scenario 2* accounts for minor damage, where partial repair or downcycling is necessary. *Scenario 3* captures worst-case deterioration, where high-energy recycling or disposal are the only viable routes. This tiered framework mirrors real practice, where uncertainty about offshore exposure requires multiple EoL contingencies^3^.

An additional layer of insight is provided through the development of component-specific inventories – documented in Appendix C - which act as material passports summarising the fate of major elements of this research’s semi-submersible FWT under each scenario. Those inventories provide the operational bridge between modelling outputs and practice. By consolidating materials, masses, recovery routes and potential applications, they make explicit what resources re-enter the economy. This transparency is valuable not only for developers and regulators but also for supply chain actors, enabling planning for secondary markets in steel, composites and mooring materials. In this study, inventories serve as the central mechanism to translate BIM-derived data into actionable recovery strategies. They also provide a baseline for future comparisons across different turbine types, decommissioning methodologies and EoL stages.

From a methodological perspective, the results demonstrate the novelty of embedding mass-based calculations into a BIM-supported workflow providing a structured environment to visualise the decommissioning process and sequence operations. In this study, although the analytical stage requires external calculations, the ability of BIM to integrate visualisation with quantification underscores its potential as a decision-support tool for complex decommissioning projects. The export to Navisworks adds a temporal dimension, illustrating deconstruction as a phased, measurable process rather than an abstract plan. This integration aligns with calls in the literature for extending BIM beyond design and construction into decommissioning, a field where structured workflows remain rare.

This study has several limitations that highlight opportunities for future research. First, the BIM model developed is simplified, focusing on major structural components rather than full engineering detail. While this ensures clarity for deconstruction sequencing, it reduces granularity in material quantification. Future work could incorporate more detailed engineering models to improve accuracy in material estimation and recovery potential. Second, the workflow is not fully dynamic, and subsequent calculations for cost, time, and CO₂e are conducted manually rather than being automatically updated across the model—this limits scalability, particularly for large wind farms. Future research could integrate automated data links between BIM and assessment tools to enable real-time updates and improved scalability. Third, the scenarios are based on secondary data and assumptions regarding end-of-life damage states. Further studies could incorporate field data from decommissioning projects or industry partnerships to validate and refine these assumptions. Finally, the analysis excludes internal mechanisms and additional parameters such as labour costs, onshore logistics, and facility costs. Future research could expand the system boundary to include these factors, providing a more comprehensive assessment of offshore wind turbine end-of-life strategies. Future research should focus on automating data links between BIM and LCA datasets to enable dynamic recalculation of costs, time, and emissions. Integration of inspection data, expansion to wind farm-scale assessments, and validation using operational decommissioning case studies would further improve accuracy and applicability. Additionally, future work will explore opportunities for making the BIM framework publicly accessible, enabling replication, benchmarking, and broader adoption by industry stakeholders.

### Sensitivity analysis

The integrated sensitivity analysis (Table 7) highlights a pronounced risk polarisation across the FWT decommissioning system, reflecting divergent stability levels between component-level processing and large-scale offshore infrastructure under the Tier-based classification framework. Composite components, specifically blades and nacelle, demonstrate high financial resilience (Tier 2) and extremely high to high environmental stability (Tier 1–2). These components exhibit relatively low cost volatility (± 16–20%) and minimal CO₂ variance (± 5%), indicating that onshore technical processing methods—such as diamond wire cutting and mechanical shredding—are largely insensitive to moderate fluctuations in operational parameters. This stability is primarily attributed to well-established industrial recycling pathways, predictable processing requirements, and linear scaling of material throughput, which collectively enhance the robustness of composite recycling strategies.

In contrast, large-scale structural components—including the tower, floating platform, and suction anchors—along with maritime logistics, exhibit low financial resilience and environmental stability (Tier 4), representing the primary sources of uncertainty within the decommissioning framework. These components exhibit substantial cost volatility (up to ± 41%) and significant environmental variability, particularly for steel-intensive assets, where emissions fluctuate asymmetrically (e.g., + 21% to − 78% across tower recycling pathways). This variability is primarily driven by the dependency on regional recycling infrastructure, specifically the choice between Electric Arc Furnace (EAF) and Blast Furnace–Basic Oxygen Furnace (BF-BOF) steel production routes, which significantly affect carbon intensity outcomes. Furthermore, offshore operational constraints—including vessel availability, weather-dependent installation windows, and subsea inspection requirements—introduce additional cost and scheduling uncertainties, particularly for logistics, where budget variation ranges from − 23% to + 68% and CO₂ emissions vary by up to + 97%.

At the system level, the integrated end-of-life (EoL) analysis reflects moderate overall stability, with total system cost variance of ± 43% and carbon variance of ± 40%. This indicates that although composite component processing remains stable and technically mature, the overall project performance is disproportionately influenced by high-risk structural and logistical elements. The sensitivity results therefore confirm that the technical feasibility of the EoL phase is robust, but project-level success is highly sensitive to infrastructure availability, steel recycling pathways, and offshore asset condition assessments.

The findings suggest that risk mitigation strategies should prioritise Tier 4 components, particularly through early asset integrity inspections, regional planning for recycling infrastructure, and contingency allocation for offshore logistics. Addressing these high-variance nodes is critical to improving both financial predictability and environmental performance, thereby enhancing the overall resilience of floating wind turbine decommissioning within a circular economy framework. The detailed about the methods selection is in Appendix D.

**Table 7:**
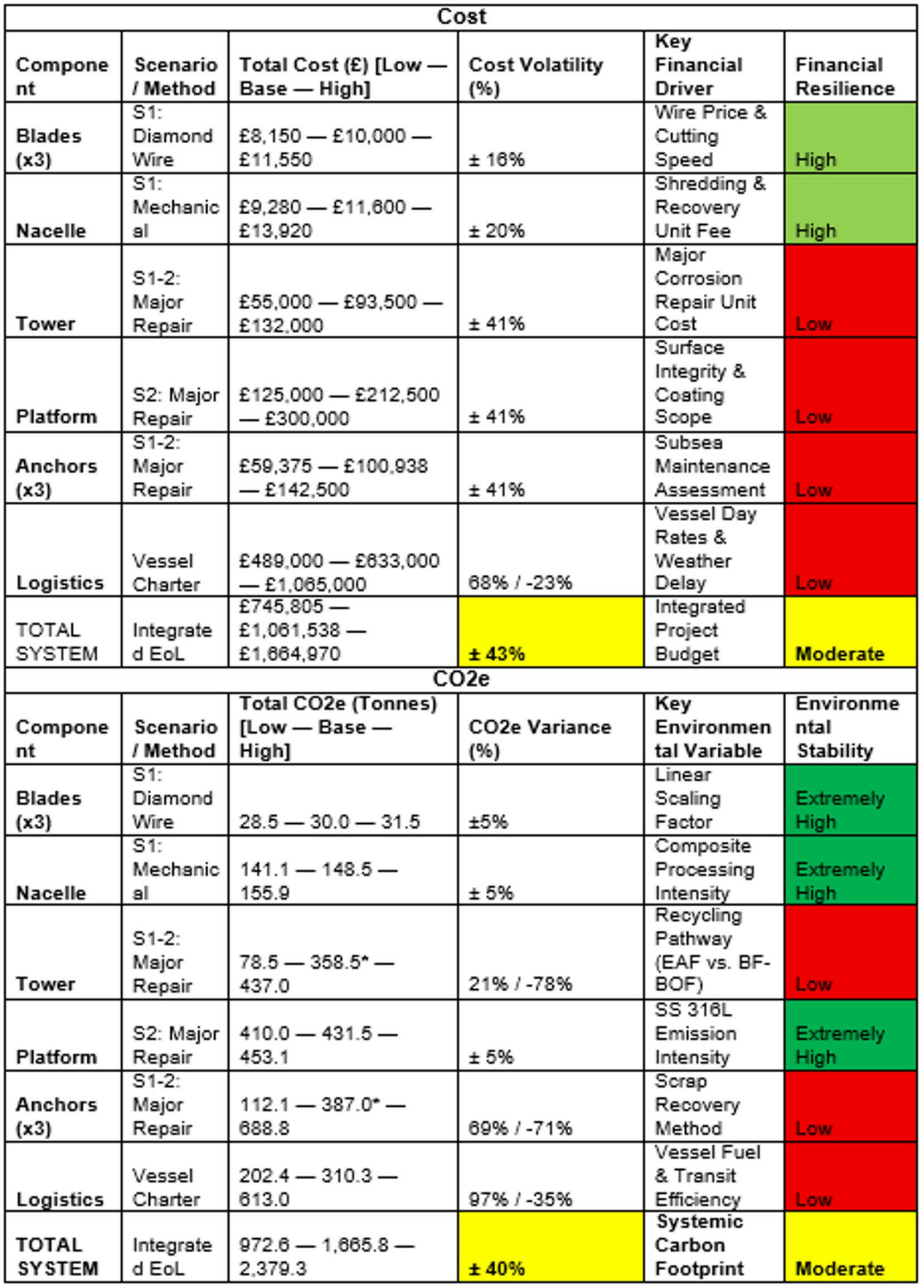

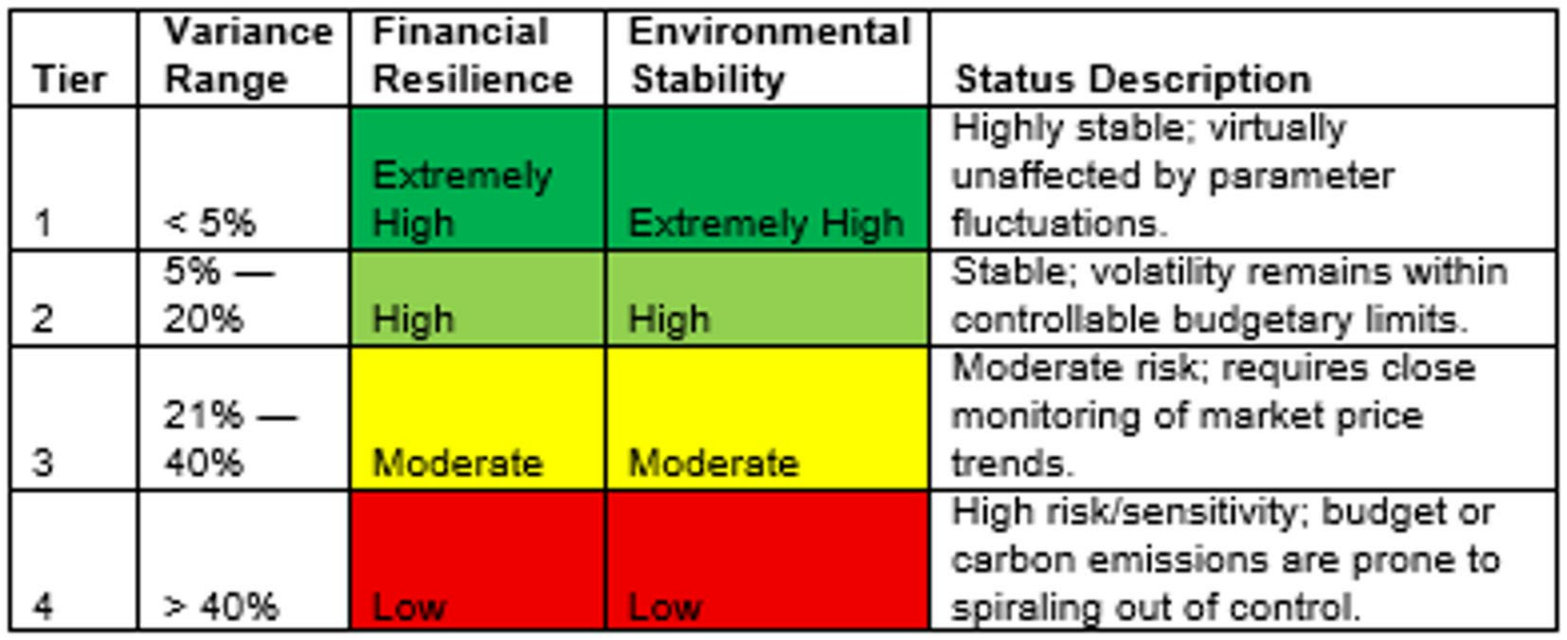
(a) Integration matrix sensitivity analysis (b) Variance Range of Financial Resilience.

*Note: Base cases for steel components represent a weighted average of EAF and BF-BOF methods to reflect infrastructure uncertainty.

## Conclusion and further research

With more than 30 GW of offshore wind capacity in Europe approaching EoL in the 2030s and an estimated 40 million tonnes of turbine materials expected globally by 2050, decommissioning represents both a challenge and an opportunity^1,3^. This study sets out to address this emerging issue using BIM as a framework for planning circular EoL strategies for semi-submersible FWTs.

The central aim — to integrate BIM-based modelling with quantitative assessment of cost, time and CO₂e emissions — has been successfully achieved. By deriving component masses directly from BIM geometry, three EoL scenarios have been developed that provide a structured framework to evaluate trade-offs between reuse, repurpose and recycling. The results confirm that *scenario 1*, assuming zero to minimal component damage, delivers the most favourable outcomes across all three parameters, while *scenario 3* consistently incurs high costs, long durations and significantly greater emissions.

Material inventories further strengthen the framework by linking digital modelling outputs to recovery strategies. By consolidating component masses, processing routes, and potential applications, the inventories demonstrate how materials can remain in circulation and provide a transparent, replicable approach for future offshore wind projects. Validation of the framework was conducted by cross-referencing BIM-derived quantities with literature-based data and industry assumptions, and by performing consistency checks across scenario outputs. This approach demonstrates the methodology’s feasibility and internal consistency, although further validation using real decommissioning projects is recommended. Overall, this study demonstrates that BIM can serve as an effective decision-support tool for circular offshore wind decommissioning, bridging the gap between digital design and sustainable EoL planning.

## Data Availability

The data that support the findings of this study are available from the corresponding author upon reasonable request.

## Appendix A

**Design specifications and calculations of FOWT components**

See Tables 8, 9 and 10.

**Table 8:** Dimensions of wind turbine components.

Component	Length (m)	Height (m)	Width (m)	Diameter (m)	Wall thickness (m)
Blades	60	–	–	–	Variable
Nacelle	16	6	5.5	–	0.02
Tower	–	90	–	6.5 (base) 3.5 (top)	0.02
Outer top buoyant columns	–	24	–	12	0.02
Outer bottom buoyant columns	–	6	–	24	0.02
Central buoyant column	–	30	–	6.5	0.02
Pontoons	15–40	–	–	1.6	0.02
Mooring lines	60	–	–	0.12	–
Suction Anchors	–	25	–	6	0.03

**Table 9:** Assigned materials to wind turbine components.

Component	Blades	Nacelle	Tower	Platform	Mooring Lines	Suction Anchors
Material	GFRP	GFRP	S355	Stainless steel 316 L	Polyester	S355

**Table 10:** Calculated masses of wind turbine components (volumes extracted from Revit).

Component	Material	Volume (m^3^)	Density (kg/m^3^)	Mass (V × D) (tn)
Blades (each)	GFRP	15	1800	27
Nacelle	GFRP	82.5	1800	148.5
Tower	S355	28	7850	220
Platform	Stainless 316 L	131.5	8000	1052
Mooring (each)	Polyester	0.7	1300	0.9
Anchors (each)	S355	14	7850	110

## Appendix B

### B1. Logistics calculations

*Vessels’ cost and duration calculations*: 3 JU Barges cost £ 48,000/day and 6 tugboats £ 15,000/day. One vessel carries the dismantled components to shore and two tow the platform to port. The vessels are rented for approximately 4 days. Therefore, total cost for JUBs and tugboats is calculated to be £633,000^46–48^.

^46,53,87^*Vessels’ CO*_*2*_*e emissions calculations*: Average JUB consumption is 6–8 tonnes / day. Assuming 7 tonnes / day and that JUB uses marine fuel diesel, for which CO_2_e emissions are 3.602 g CO_2_e per gram marine diesel consumed^46,53,87^. Therefore, total CO_2_e emissions per JUB per day are approximately 25.2 tonnes. Each JUB generates 100.8 tonnes CO_2_e over the 4-day period, therefore the total JUB emissions are calculated to be around 302 tonnes. According to a study made for hybrid tugboats, total CO_2_e emissions are estimated to be 165 kg CO_2_e / hour^54^. For our case, the tugboats are operating for 8 h of the transportation time, so each tugboat emits approximately 1.320 kg CO_2_e for the whole transportation operation and the total CO_2_e emissions for all 6 tugboats are 7.92 tonnes.

### B2. Blades calculations

**Scenario 1**

*Duration*: Cutting GFRP blades with diamond wiring requires 6.5 min per meter^55^. The narrowest part is approximately 1.80 m and the widest is 4.90 m. On average, the blade is 3.40 m thick. So, each cut is completed in about 22 min. Assuming 60 cuts per blade (blades are 60 m–1 m per cut) it takes 22 h per blade which is around 3 days (72 h) for 3 blades.

*Cost*: Processing per blade costs £2,500 and diamond wiring costs £8 - £50 per tonne^56^. Therefore, assuming an average of £30 per tonne, wiring costs £810 per blade. This adds up to £10,000 for this scenario.

*CO*_*2*_*e*: ADDIN ZOTERO_ITEM CSL_CITATION {“citationID”:“8cKaZzDi”,“properties”:{“formattedCitation”:“\\super 6\\nosupersub{}”,“plainCitation”:“6”,“noteIndex”:0},“citationItems”:[{“id”:685,“uris”:[“http://zotero.org/users/13337376/items/H9J87ZT6”],“itemData”:{“id”:685,“type”:“report”,“publisher”:“World Steel Association”,“title”:“World Steel in Figs. 2025”,“URL”:“https://worldsteel.org/data/world-steel-in-figures/world-steel-in-figures-2025/“,“author”:[{“literal”:“World Steel Association”}]}}],“schema”:“https://github.com/citation-style-language/schema/raw/master/csl-citation.json”} ^54^Approximately 35 tonnes for processing three 71-meter blades are produced according to a study^58^. Based on that, it is estimated that 30 tonnes for this report’s 60-meter blades are generated.

**Scenario 2**

*Cost*: Mechanical repairing costs £78 per tonne^59^. Therefore, for all three blades the total repairing cost is approximately £6,500.

*CO*_*2*_*e*: Approximately 50 tonnes CO_2_ for recycling three 71-meter blades with shredding are produced according to a study^58^. Based on that, it is estimated that 43 tonnes for this report’s 60-meter blades are generated.

**Scenario 3**

*Cost*: Pyrolysis costs £460 per tonne^65^. Therefore, for all three blades the total pyrolysis cost is approximately £37,500. Solvolysis costs £3,000 per tonne^68^. Therefore, for all three blades the total solvolysis cost is approximately £243,000.

*CO*_*2*_*e*: Approximately 268 tonnes CO_2_ for recycling three 71-meter blades with pyrolysis are produced according to a study^58^. Based on that, it is estimated that 225 tonnes for this report’s 60-meter blades are generated (Table 11).

**Table 11:** Blade scenarios in EoL.

Scenario	Description	Potential damage	CE Rs feasibility	EoL processes	Risk/uncertainty
1	Perfect condition	–	Immediate reuse/repurpose	Inspect, cut down for reuse/repurpose	Misclassification in inspection could overstate reuse potential
2	Minor damage	Minor surface stripping, edge cracking	Possible reuse/repurpose	Mechanical repair	Residual fatigue may limit safe repurpose
3	Major damage	Leading-edge erosion, severe cracking, surface peeling	Recycling	Mechanical recycling (shredding), chemical recycling (solvolysis), thermal recycling (pyrolysis)	Fibre quality and market demand uncertain

Scenario 1 assumes structural integrity and allows direct repurposing, but its success depends on reliable inspection practices to avoid safety risks. Scenario 2 represents cases of moderate degradation where repair may restore functionality, though the uncertainty lies in balancing repair cost against limited extension of service life. Scenario 3 accounts for severely degraded blades where only recycling is viable, yet this pathway carries the greatest uncertainty due to inconsistent fibre recovery quality, high process costs and limited end-markets.

### B3. Nacelle calculations

**Scenario 3**

*Cost*: Mechanical shredding costs £78 per tonne^59^. Therefore, for the nacelle the total recycling cost is approximately £11,600. Pyrolysis costs £460 per tonne^65^. Therefore, for the nacelle the total recycling cost is approximately £68,300. Solvolysis costs £3,000 per tonne^68^. Therefore, for the nacelle the total recycling cost is approximately £445,500 (Table 12).

**Table 12:** Nacelle scenarios in EoL.

Scenario	Description	Potential damage	CE Rs feasibility	EoL processes	Risk/uncertainty
1	Perfect condition	–	Immediate reuse/repurpose	Inspect, remove, reuse / repurpose	Hidden micro-defects or fatigue not always detectable
2	Minor damage	Minor surface wear, cracked patches, holes	Minor repairs for reuse/repurpose	Selective repair	Residual wear may shorten extended service life
3	Major damage	Extensive cracking, compromised shell	Recycling	Mechanical recycling (shredding), chemical recycling (solvolysis), thermal recycling (pyrolysis)	Fibre recovery quality and cost-effectiveness uncertain

Scenario 1 assumes structural integrity, enabling reuse or repurposing with minimal intervention, though confidence depends on accurate inspection methods. Scenario 2 covers moderate damage where localised repairs could restore function, but uncertainty arises from the durability of repairs relative to costs. Scenario 3 reflects severe degradation where recycling is the only option, yet this pathway carries significant uncertainty because nacelle composites and mixed materials complicate recycling, with variable recovery quality and high processing costs.

### B4. Tower calculations

**Scenario 1 & 2**

*Cost*: Repairing minor corrosion spots costs £25–£100 per m^[279^.The total surface area of the tower is 1456 m^2^. Assuming an average repairing price of £62.5 per m^2^ and that 15% of the tower’s surface area (220 m^2^) needs to be repaired, the total repairing cost for minor damage will be approximately £14,000. Repairing major corrosion spots costs £250–£600 per m^[279^. Assuming an average repairing price of £425 per m^2^, the total repairing cost for major damage will be approximately £93,000.

**Scenario 3**

*CO*_*2*_*e*: Electric Arc Furnace (EAF) recycling method produces 0.357 tonnes of CO_2_e per tonne of recycled steel. Therefore, recycling the tower with EAF produces approximately 78.5 tonnes CO_2_e. Blast Furnace – Basic Oxygen Furnace (BF – BOF) recycling method produces 1.987 tonnes of CO_2_e per tonne of recycled steel. Therefore, recycling the tower with BF - BOF produces approximately 437 tonnes CO_2_e^14^ (Table 13).

**Table 13:** Tower scenarios in EoL.

Scenario	Description	Potential damage	CE Rs feasibility	EoL processes	Risk/uncertainty
1	Perfect condition	–	Immediate reuse/repurpose	Inspect, remove, reuse/repurpose	Undetected fatigue or micro-cracks could compromise safety
2	Minor damage	Minor corrosion spots, small distortions	Minor repairs for limited reuse/repurpose	Selective repair, Welding	Weld quality introduces uncertainty
3	Major damage	Major corrosion spots, fatigue, severe deformation	Recycling	Electric Arc Furnace (EAF), Blast Furnace – Basic Oxygen Furnace (BF-BOF)	Uncertain steel market demand

Scenario 1 assumes sound condition confirmed by inspection, supporting direct reuse in other infrastructure applications, though risks remain if hidden fatigue is missed. Scenario 2 reflects moderate degradation where welding or selective repair can extend service life, yet uncertainties lie in repair quality and whether residual strength justifies repurpose. Scenario 3 represents severe degradation where recycling is the only feasible route. While steel recycling is mature, uncertainties relate to process selection (EAF vs. BF-BOF) and the variability across markets.

### B5. Platform calculations

**Scenario 2**

*Cost*: Repairing minor corrosion spots costs £25–£100 per m^[2 79^. The total surface area of the platform is approximately 6750 m^2^. Assuming an average repairing price of £62.5 per m^2^ and that 15% of the platform’s surface area (1050 m^2^) needs to be repaired, the total repairing cost for minor damage will be approximately £66,000. Repairing major corrosion spots costs £250–£600 per m^[2 79^. Assuming an average repairing price of £425 per m^2^, the total repairing cost for major damage will be approximately £212,500.

**Scenario 3**

*CO*_*2*_*e*: Recycling SS 316 L produces 0.41 tonnes CO_2_e per tonne of recycled steel^15^. Therefore, recycling the platform produces approximately 431.5 tonnes CO_2_e (Table 14).

**Table 14:** Platform scenarios in EoL.

Scenario	Description	Potential damage	CE Rs feasibility	EoL processes	Risk/uncertainty
1	Perfect condition	–	Immediate reuse/repurpose	Inspect, remove, reuse/repurpose	Hidden fatigue or corrosion may be overlooked
2	Minor damage	Small crack, shallow pitting	Minor repairs for limited reuse/repurpose	Selective repair, Welding	Risk of corrosion recurrence in repurposed structures
3	Major damage	Deep corrosion, severe cracking, excessive fatigue	Recycling	Melting and refining for new 316 L products	Uncertain quality retention

Scenario 1 assumes sound condition validated by inspection, enabling immediate reuse or repurposing, though risks exist if micro-cracking or hidden corrosion is missed. Scenario 2 represents limited degradation, where welding may restore usability, yet uncertainty lies in the long-term durability of repairs and the likelihood of recurring corrosion in repurposed applications. Scenario 3 reflects severe degradation where only recycling is viable. While stainless steel recycling is mature, uncertainties persist around the high energy demand of refining and the ability to maintain steel quality during remelting.

### B6. Mooring lines calculations

See Table 15.

**Table 15:** Mooring lines scenarios in EoL.

Scenario	Description	Potential damage	CE Rs feasibility	EoL processes	Risk/uncertainty
1	Perfect condition	–	Reuse in similar settings	Inspect, remove, reuse	Hidden internal wear or fatigue may still reduce performance
2	Minor damage	Frayed sections, minor wear	Remanufacture/downcycle	Use as secondary rope	Lack of residual strength may limit safe reuse
3	Major damage	Severe ftigue/fracture, UV degradation, loss of strength	Limited recycling/disposal	Mechanical shredding, pyrolysis with limited application	Uncertain outcomes

Scenario 1 assumes intact ropes suitable for reuse in future marine projects, though internal fibre fatigue could be overlooked without advanced inspection. Scenario 2 accounts for moderate wear such as fraying, where lines may be cut and repurposed for secondary applications, yet uncertainty lies in verifying residual strength and durability. Scenario 3 represents severe degradation, limiting options to energy-intensive recycling or disposal. Here, uncertainty is greatest as recycling technologies for synthetic fibres remain immature and secondary markets are limited.

### B7. Suction anchors calculations

**Scenario 1–2**

*Cost*: Repairing minor corrosion spots costs £25 - £100 per m^[279^. The total surface area of all three anchors is approximately 1,583 m^2^. Assuming an average repairing price of £62.5 per m^2^ and that 15% of the anchors’ surface area (237.5 m^2^) needs to be repaired, the total repairing cost for minor damage will be approximately £15,000. Repairing major corrosion spots costs £250 - £600 per m^[279^. Assuming an average repairing price of £425 per m^2^, the total repairing cost for major damage will be approximately £101,000.

**Scenario 3**

*CO*_*2*_*e*: Electric Arc Furnace (EAF) recycling method produces 0.357 tonnes of CO_2_e per tonne of recycled steel. Therefore, recycling the anchors with EAF produces approximately 118 tonnes CO_2_e. Blast Furnace – Basic Oxygen Furnace (BF – BOF) recycling method produces 1.987 tonnes of CO_2_e per tonne of recycled steel. Therefore, recycling the anchors with BF - BOF produces approximately 656 tonnes CO_2_e^14^ (Table 16).

**Table 16:** Suction Anchors scenarios in EoL.

Scenario	Description	Potential damage	CE Rs feasibility	EoL processes	Risk/uncertainty
1	Perfect condition	–	Reuse in similar settings	Inspect, remove, reuse	Hidden micro-cracks or fatigue from seabed loading may be missed
2	Minor damage	Localised corrosion, minor weld damage	Repair for further reuse	Spot repair, surface treatment, repurpose	Uncertain long-term durability
3	Major damage	Heavy corrosion, major cracks	Recycling	Electric Arc Furnace (EAF), Blast Furnace – Basic Oxygen Furnace (BF-BOF)	Uncertain steel market demand

Scenario 1 assumes structural integrity confirmed by inspection, allowing direct reuse or repurposing in offshore or marine projects, though hidden fatigue may compromise reliability if overlooked. Scenario 2 accounts for moderate degradation such as surface corrosion, where repair or limited repurposing is possible, but uncertainties remain regarding the long-term performance of treated structures. Scenario 3 reflects severe degradation where only recycling is viable. While steel recycling is mature and reliable, uncertainties relate to process selection (EAF vs. BF-BOF) and the variability across markets.

## Appendix C

**Inventories**

See Tables 17, 18 and 19.

**Table 17:** Scenario 1 inventory at EoL.

Component	Material	Quantity (t)	Route/process	CE principle	EoL application
Blades	GFRP	27	Cutting with diamond saws and repurpose	Repurpose	Playgrounds, bridges, benches
Nacelle	GFRP	148.5	Direct reuse	Reuse	Off-grid energy enclosures
Tower	S355	220	Direct repurpose	Repurpose	Telecom and water towers, storage silos, vertical farms
Platform (pontoons and buoyant columns)	SS 316 L	1,052	Plasma cutting and repurpose	Repurpose	Aquaculture cages, floating solar panels, bridges
Mooring lines	Polyester	0.9	Direct reuse	Reuse	Similar marine projects
Suction anchors	S355	330	Direct reuse	Reuse	Similar marine projects

**Table 18:** Scenario 2 inventory at EoL.

Component	Material	Quantity (t)	Route/process	CE principle	EoL application
Blades	GFRP	27	Possible repurpose after mechanical repairing	Repurpose	Playgrounds, bridges, benches
Nacelle	GFRP	148.5	Repurpose after minor selective repairs	Repurpose	Off-grid energy enclosures
Tower	S355	220	Possible repurpose after welding repairing	Repurpose	Telecom and water towers, storage silos, vertical farms
Platform (pontoons and buoyant columns)	SS 316 L	1,052	Possible repurpose after welding repairing	Repurpose	Aquaculture cages, floating solar panels, bridges
Mooring lines	Polyester	0.9	Possible reuse / repurpose after remanufacturing or downcycling	Reuse / Repurpose	Secondary rope
Suction anchors	S355	330	Possible reuse / repurpose after welding repairing	Reuse / Repurpose	Similar marine projects

**Table 19:** Scenario 3 inventory at EoL.

Component	Material	Quantity (t)	Route/process	CE principle	EoL application
Blades	GFRP	27	Shredding, pyrolysis, solvolysis	Recycling	Fibres recovery, concrete feedstock, other GFRP products
Nacelle	GFRP	148.5	Shredding, pyrolysis, solvolysis	Recycling	Fibres recovery, concrete feedstock, other GFRP products
Tower	S355	220	Melting via EAF or BF - BOF	Recycling	High-grade, low carbon steel
Platform (pontoons and buoyant columns)	SS 316 L	1,052	Melting for recycling	Recycling	Refining for new 316 L products
Mooring lines	Polyester	0.9	Limited recycling	Recycling	Disposal, other polyester products
Suction anchors	S355	330	Melting via EAF or BF - BOF	Recycling	High-grade, low carbon steel

## Appendix D. Data selection of the sensitivity analysis

The integration of component-specific methods within the Master Matrix represents a deliberate strategy to enhance operational realism and reflect the heterogeneous nature of floating wind turbine decommissioning. By selecting technically mature processes and robust, data-rich sources as baseline assumptions, the analysis ensures that the modelling framework reflects current industrial practices rather than theoretical or emerging technologies. This methodological approach enables the identification of a pronounced risk polarisation effect across the system.

Specifically, the analysis differentiates between inherently stable technical nodes, such as blade and nacelle processing, which rely on established onshore recycling pathways, and highly volatile operational dependencies, including offshore logistics, large-scale structural component handling, and regional steel recycling infrastructure. These latter elements exhibit greater sensitivity to external factors such as weather windows, vessel availability, and regional variations in recycling technologies (e.g., EAF versus BF–BOF routes).

By capturing these variations, the Master Matrix framework provides a more nuanced and robust decision-support tool for stakeholders. Compared with a homogenised single-method model, this approach better reflects real-world operational constraints, improves risk identification, and supports more informed planning for cost control, carbon reduction, and the implementation of a circular economy in FWT decommissioning (Table 20).

**Table 20:** Data selection of the sensitivity analysis.

Component	Selected base method	Primary rationale for selection
Blades	Scenario 1 (Diamond Wire)	High data granularity from recent literature represents the current best practice for high-quality composite material recovery
Nacelle	S-N1 (Mechanical Shredding)	An established industrial standard which provides the most reliable cost baseline
Tower	Major Corrosion Repair	Adopts a conservative risk-management approach to account for potential structural degradation in harsh offshore environments
Platform	Major Corrosion Repair	Selected due to the massive surface area (6,750 m^2^) which makes surface integrity the dominant financial driver
Anchors	Weighted Average (EAF/BF-BOF)	Reflects the systemic uncertainty of regional steel recycling infrastructure and its associated carbon intensity
Logistics	Standard Vessel Charter	Based on median market day rates and typical North Sea weather window availability

## Abbreviations

AEC: Architecture, Engineering and Construction
BF-BOF: Blast Furnace – Basic Oxygen Furnace
BIM: Building information modelling
CE: Circular economy
DWM: Demolition waste management
EAF: Electric arc furnace
EoL: End-of-life
FWT: Floating wind turbine
GFRP: Glass–fiber reinforced plastic
HAZ: Heat-affected zones
JUB: Jack-up barge
LCA: Life-cycle assessment
SS: Stainless steel
